# Eosinophils support adipocyte maturation and promote glucose tolerance in obesity

**DOI:** 10.1038/s41598-018-28371-4

**Published:** 2018-07-02

**Authors:** Eun-Hui Lee, Michal Itan, Jinsun Jang, Hyeon-Jung Gu, Perri Rozenberg, Melissa K. Mingler, Ting Wen, Jiyoung Yoon, Shi-Young Park, Joo Young Roh, Cheol Soo Choi, Woo-Jae Park, Ariel Munitz, YunJae Jung

**Affiliations:** 1 Department of Microbiology, School of Medicine, Gachon University, Incheon, 21999 Korea; 2 Department of Clinical Microbiology and Immunology, The Sackler School of Medicine, Tel-Aviv University, Ramat Aviv, 69978 Israel; 3 Department of Dermatology, Gachon Gil Medical Center, School of Medicine, Gachon University, Incheon, 21565 Korea; 4 Division of Allergy and Immunology, Cincinnati Children’s Hospital Medical Center, University of Cincinnati College of Medicine, Cincinnati, OH 45229 USA; 5 Department of Molecular Medicine, Lee Gil Ya Cancer and Diabetes Institute, School of Medicine, Gachon University, Incheon, 21999 Korea; 6 Korea Mouse Metabolic Phenotyping Center, Lee Gil Ya Cancer and Diabetes Institute, School of Medicine, Gachon University, Incheon, 21999 Korea; 7 Department of Biochemistry, School of Medicine, Gachon University, Incheon, 21999 Korea; 8 Gachon Advanced Institute for Health Science & Technology, Gachon University, Incheon, 21999 Korea

## Abstract

Accumulating data have indicated a fundamental role of eosinophils in regulating adipose tissue homeostasis. Here, we performed whole-genome RNA sequencing of the small intestinal tract, which suggested the presence of impaired lipid metabolism in eosinophil-deficient ΔdblGATA mice. ΔdblGATA mice fed a high-fat diet (HFD) showed reduced body fat mass, impaired enlargement of adipocytes, decreased expression of adipogenic genes, and developed glucose intolerance. HFD induced accumulation of eosinophils in the perigonadal white adipose tissue. Concordantly, adipocyte-differentiated 3T3-L1 cells promoted the migration of eosinophils through the expression of CCL11 (eotaxin-1) and likely promoted their survival through the expression of interleukin (IL)-3, IL-5, and granulocyte-macrophage colony-stimulating factor. HFD-fed ΔdblGATA mice showed increased infiltration of macrophages, CD4^+^ T-cells, and B-cells, increased expression of interferon-γ, and decreased expression of IL-4 and IL-13 in white adipose tissue. Interferon-γ treatment significantly decreased lipid deposition in adipocyte-differentiated 3T3-L1 cells, while IL-4 treatment promoted lipid accumulation. Notably, HFD-fed ΔdblGATA mice showed increased lipid storage in the liver as compared with wild-type mice. We propose that obesity promotes the infiltration of eosinophils into adipose tissue that subsequently contribute to the metabolic homeostasis by promoting adipocyte maturation.

## Introduction

Eosinophils have been considered as destructive cells involved in T helper cell type (Th) 2 immune responses in parasitic infections or allergic diseases^1^. However, accumulating evidence has indicated additional roles for eosinophils. For example, eosinophils reside in several organs including the gastrointestinal tract^2,3^ and adipose tissue and contribute to metabolic homeostasis^4,5^. Adipose tissue eosinophils secrete interleukin (IL)-4 and induce the polarization of white adipose tissue (WAT) macrophages into alternatively-activated macrophages, which support glucose tolerance by regulating local catecholamine stores in the microenvironment through the import of catecholamines produced by nerve cells^4,6,7^. Despite recent advances, the roles of eosinophils in the adipose tissue and their effects on adipocyte function remain incompletely understood.

Obesity and its associated metabolic disorders are serious health problems worldwide^8^. However, obesity is not necessarily an adverse metabolic condition when the excess fat is stored in adipose tissue that responds to insulin^9^. Adipose tissue regulates energy homeostasis through the storage of excess calories and the secretion of adipocyte-derived secretory proteins such as leptin, adiponectin, and resistin^10,11^. However, excessive caloric intake induces the overexpansion of adipocytes, which results in inflammatory responses within adipose tissue^12^. Obesity-related metabolic dysfunctions are associated with an excessive infiltration of immune cells and chronic inflammation in adipose tissue^13,14^.

Here, we used eosinophil-deficient ∆dblGATA and wild-type (WT) mice to investigate the roles of eosinophils in obesity, adipose tissue maturation, and associated metabolic responses. We performed whole-genome RNA sequencing of the small intestinal tract, which suggested the presence of a defective lipid metabolism in the absence of eosinophils. Furthermore, ∆dblGATA mice fed a high-fat diet (HFD) gained less weight and showed reduced body fat, an impaired enlargement of adipocytes, a decreased expression of adipogenic genes, and a more severe glucose intolerance than the WT group. Thus, we hypothesized that the inability to appropriately expand adipose tissue underlies insulin resistance in ΔdblGATA mice. We found that adipocyte-differentiated 3T3-L1 cells promoted the migration of eosinophils through the expression of CC chemokine ligand (CCL) 11 (eotaxin-1) and promoted their survival through the expression of IL-3, IL-5, and granulocyte-macrophage colony-stimulating factor (GM-CSF). Accordingly, the WAT of HFD-fed WT mice showed an increased infiltration of eosinophils with upregulated CCL11. Compared to WT mice, ∆dblGATA mice showed significantly increased populations of pro-inflammatory immune cells in their WAT. The WAT of HFD-fed ∆dblGATA mice also showed an increased expression of the Th1 cytokine interferon (IFN)-γ and a decreased expression of the Th2 cytokines IL-4 and IL-13. The cytokine expression profile of the WAT of ∆dblGATA mice negatively correlated with adipocyte maturation as demonstrated by attenuated lipid storage in IFN-γ-treated 3T3-L1 cells, which was opposed by IL-4 treatment. Notably, HFD-fed ΔdblGATA mice showed increased lipid storage in the liver as compared with WT mice, suggesting that insulin resistance was induced by an excessive accumulation of lipid in non-adipose tissues. Thus, we propose that cross-talk between adipocytes and eosinophils promotes metabolic homeostasis by supporting the infiltration of eosinophils into adipose tissue and maintaining the microenvironment of adipose tissue to favour adipocyte maturation.

## Results

### Eosinophil-deficient mice showed impaired glucose tolerance and a decreased expression of genes involved in energy metabolism

**Figure 1:**
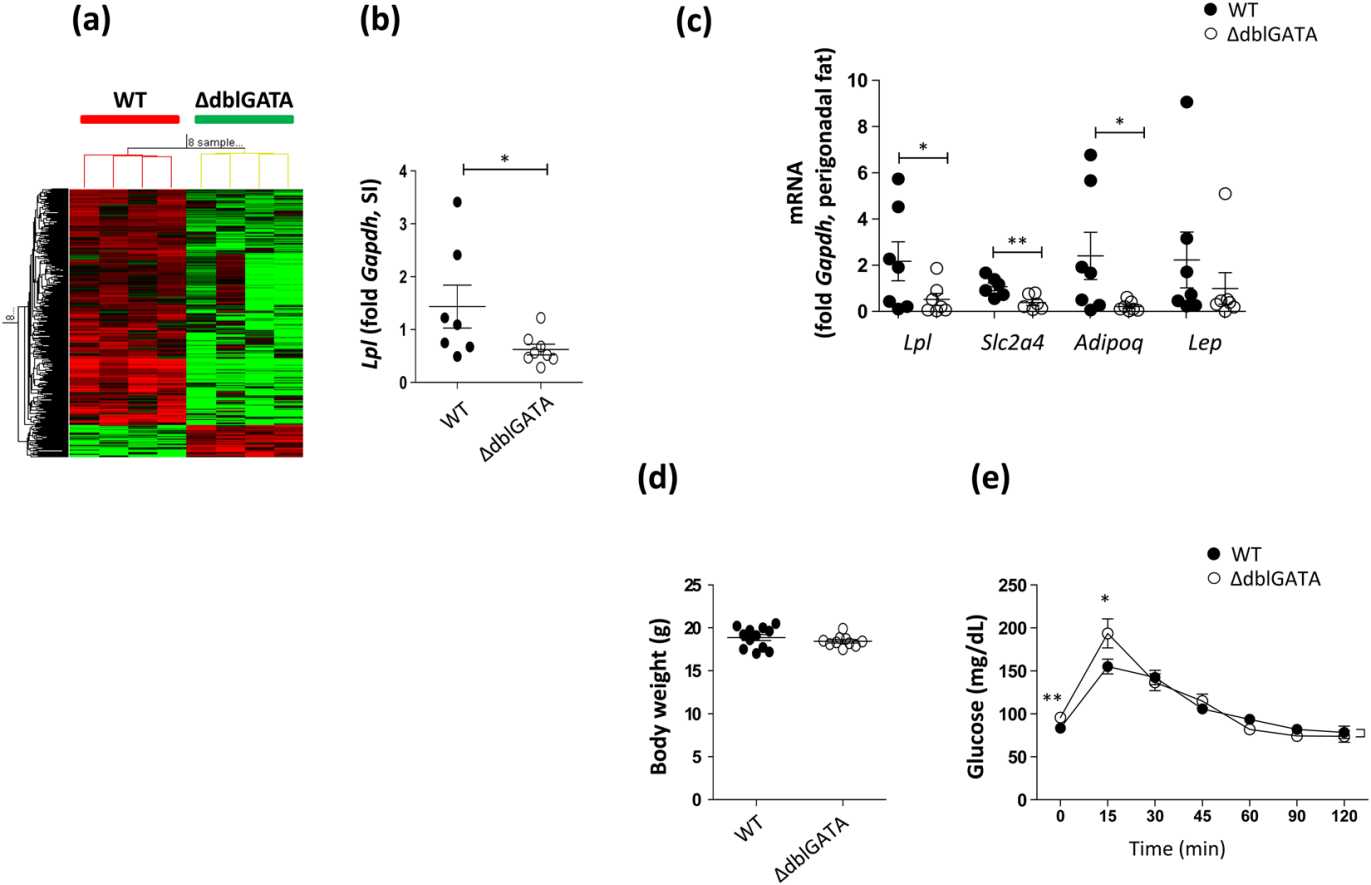
ΔdblGATA mice showed impaired glucose tolerance and a decreased expression of genes involved in energy metabolism. (**a**) RNA sequencing data obtained from the small intestine (SI) of 8–10-week-old wild-type (WT) and ΔdblGATA mice. The expression of 379 downregulated and 52 upregulated genes was observed in ΔdblGATA mice with a fragments per kilobase of exon per million fragments mapped value >0.1 in at least 1 out of 8 analysed samples. Red indicates a high expression and green indicates a low expression. (**b**) Real-time PCR analysis of *Lpl* in the SI. *p < 0.05 (Student’s *t*-test). (**c**) mRNA expression levels of adipogenic and adipokine genes in the perigonadal fat of 8–10-week-old male mice fed with a chow diet. *p < 0.05, **p < 0.01 (Student’s *t*-test for *Lpl*, *Slc2a4*, and *Adipoq*, Mann-Whitney test for *Lep*). (**d**) Body weight of 8–10-week-old male mice fed with a chow diet. (**e**) Glucose tolerance test of 8–10-week-old male mice fasted for 16 h (*n* = 10–12 mice/group). *p < 0.05, **p < 0.01 (Student’s *t*-test). Graphs show the mean ± standard error of the mean.

Under physiological conditions, eosinophils primarily reside in the small intestine, where they account for a substantial fraction (e.g., 20–30%) of the cellular population^2,3^. Therefore, the significant downregulation of genes in the small intestine of ΔdblGATA mice may indicate a role for eosinophils in regulating certain biological responses. Through the whole-genome RNA sequencing of the small intestine, we observed 379 downregulated and 52 upregulated genes in the small intestine of ΔdblGATA mice (Fig. 1a, Tables S1 and S2) and the expression of the top 10 downregulated genes was validated by real-time PCR (Fig. S1). Of note, genes associated with lipid metabolism, such as *Retnlg*, *Alox15*, and *Drd2*^15–17^ were included in the top 10 downregulated genes (Table S1). Additionally, a GO analysis of the downregulated genes and visualization of a functionally grouped network using the ClueGO plugin^18^ suggested a defect in lipase activity (Table S3 and Fig. S2) and a significant decrease in the expression of *Lpl* was observed (Fig. 1b). A decreased expression of *Lpl* and other lipogenic genes (*Slc2a4* and *Adipoq*) was also observed in the perigonadal WAT of ΔdblGATA mice (Fig. 1c). Although the body weight of 8–10-week-old ΔdblGATA mice was not different from that of WT mice (Fig. 1d), ΔdblGATA mice showed a significantly higher glucose level at the early time point of GTT (Fig. 1e). Our observations suggested that eosinophils regulate lipid metabolism and energy homeostasis.

### Eosinophil-deficient mice gained less weight and perigonadal fat mass and displayed impaired glucose tolerance on a HFD

**Figure 2:**
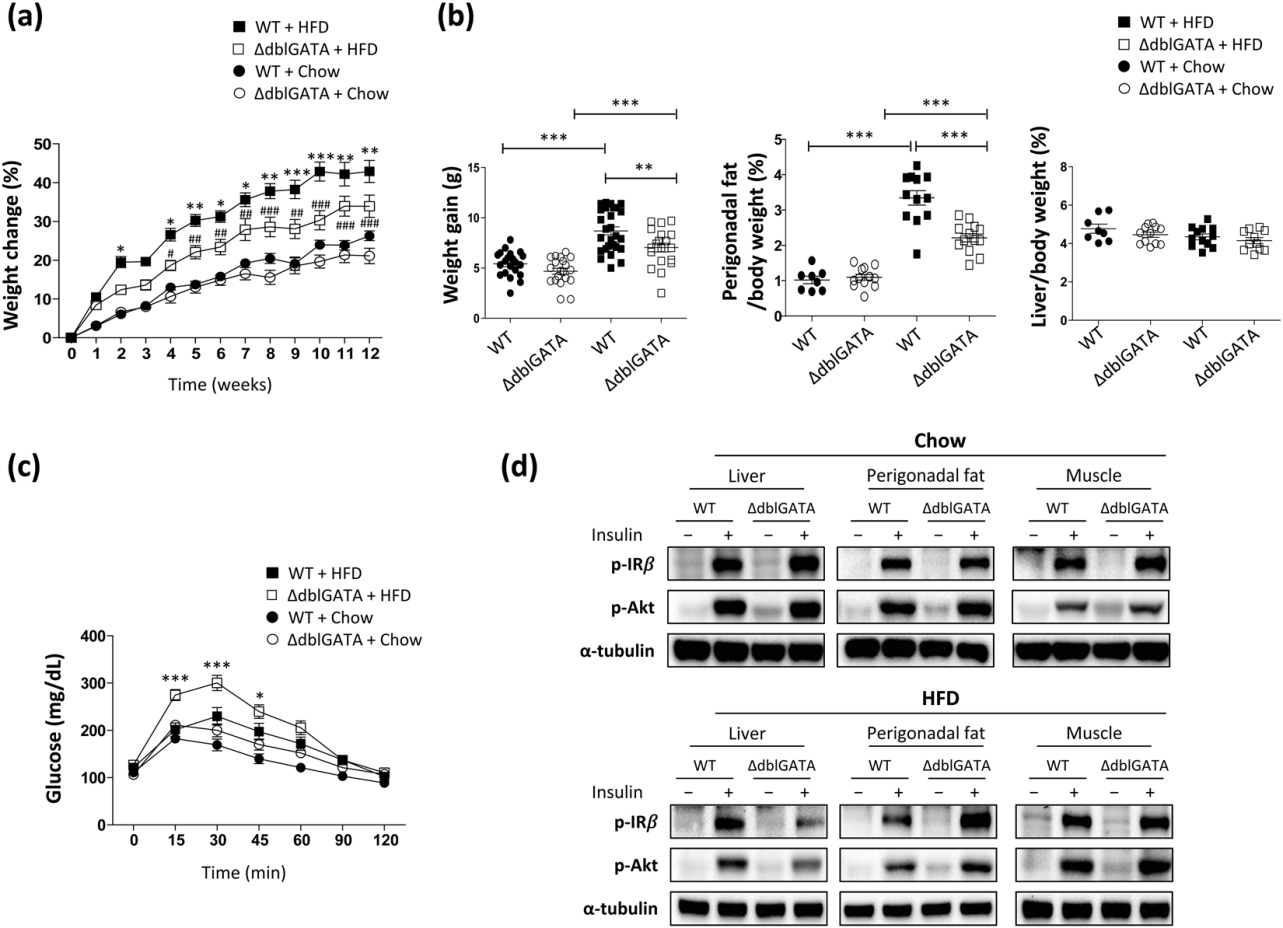
ΔdblGATA mice gained less weight and body fat and displayed impaired glucose tolerance on a high-fat diet (HFD). Wild-type (WT) and ΔdblGATA male mice were fed a HFD or chow diet for 12 weeks. (**a**) Weight change during feeding with a HFD or chow diet (*n* = 20–27 mice/group). *p < 0.05, **p < 0.01, ***p < 0.001 (two-way ANOVA), WT + HFD vs. ΔdblGATA + HFD. ^#^*p* < 0.05, ^##^*p* < 0.01, ^###^*p* < 0.001 (two-way ANOVA), ΔdblGATA + Chow vs. ΔdblGATA + HFD. (**b**) Body weight gain, perigonadal fat/body mass, and liver/body mass on a HFD or chow diet. **p < 0.01, ***p < 0.001 (one-way ANOVA). (**c**) Glucose tolerance test of the indicated mice fasted for 16 h (*n* = 13–15 mice/group). *p < 0.05, ***p < 0.001 (two-way ANOVA), WT + HFD vs. ΔdblGATA + HFD. (**d**) Western blots of insulin-stimulated phosphorylation of insulin receptor (IR) and protein kinase B (Akt) in the liver, perigonadal fat, and skeletal muscle. The data are representative of two independent experiments. Graphs show the mean ± standard error of the mean.

Excess caloric intake induces expansion of fat mass and accelerates development of insulin resistance^10^. To further examine the role of eosinophils in adipose tissue expansion and energy homeostasis, we fed 8–10-week-old male WT and ΔdblGATA mice with a HFD for 12 weeks and monitored their body weights weekly. The HFD induced a significant weight gain of ΔdblGATA mice compared to chow diet-fed ΔdblGATA mice (Fig. 2a). However, ΔdblGATA mice showed an approximately 20% lower body weight than WT mice after 12 weeks of a HFD (Fig. 2a,b). Consistent with their lesser weight gain, ΔdblGATA mice fed a HFD had significantly less perigonadal fat mass than WT mice (Figs 2b and S3a). Differences in liver mass were not apparent among the analysed groups of mice (Figs 2b and S3a). Although ΔdblGATA mice showed less weight gain under a HFD than their WT counterparts, their ability to normalize blood glucose in the GTT was significantly impaired (Fig. 2c). HFD-fed ΔdblGATA mice showed significantly higher blood glucose levels until 30 min after insulin administration (ITT, Fig. S3b); however, the slopes of the ITT curves between WT and ΔdblGATA appear to overlap and blood insulin levels did not differ between WT and ΔdblGATA mice (Fig. S3c). To assess the molecular basis of insulin resistance observed in HFD-ΔdblGATA mice, we investigated insulin receptor signalling after infusion of insulin through the portal vein. Insulin receptor (IR) tyrosine phosphorylation and protein kinase B (Akt) phosphorylation were reduced in the livers, but not in WAT or skeletal muscle from HFD-fed ΔdblGATA mice compared to in their WT counterparts (Fig. 2d). Phosphorylation of either IR or Akt was comparable between WT and ΔdblGATA mice fed a chow diet (Fig. 2d). Taken together, our observations indicate that hepatic insulin resistance was induced in ΔdblGATA mice by HFD feeding.

Although ΔdblGATA mice fed with HFD showed increased level of serum free fatty acid than WT mice (Fig. S4a), it is unlikely that ΔdblGATA mice have defect in energy utilization considering the comparable serum triglyceride and stool fat content of the HFD-fed WT and ΔdblGATA mice (Fig. S4a,b). Energy intake and expenditure were also comparable between HFD-fed WT and ΔdblGATA mice (Fig. S4c).

### Eosinophil-deficient mice showed an impaired maturation of perigonadal adipocytes on a HFD

**Figure 3:**
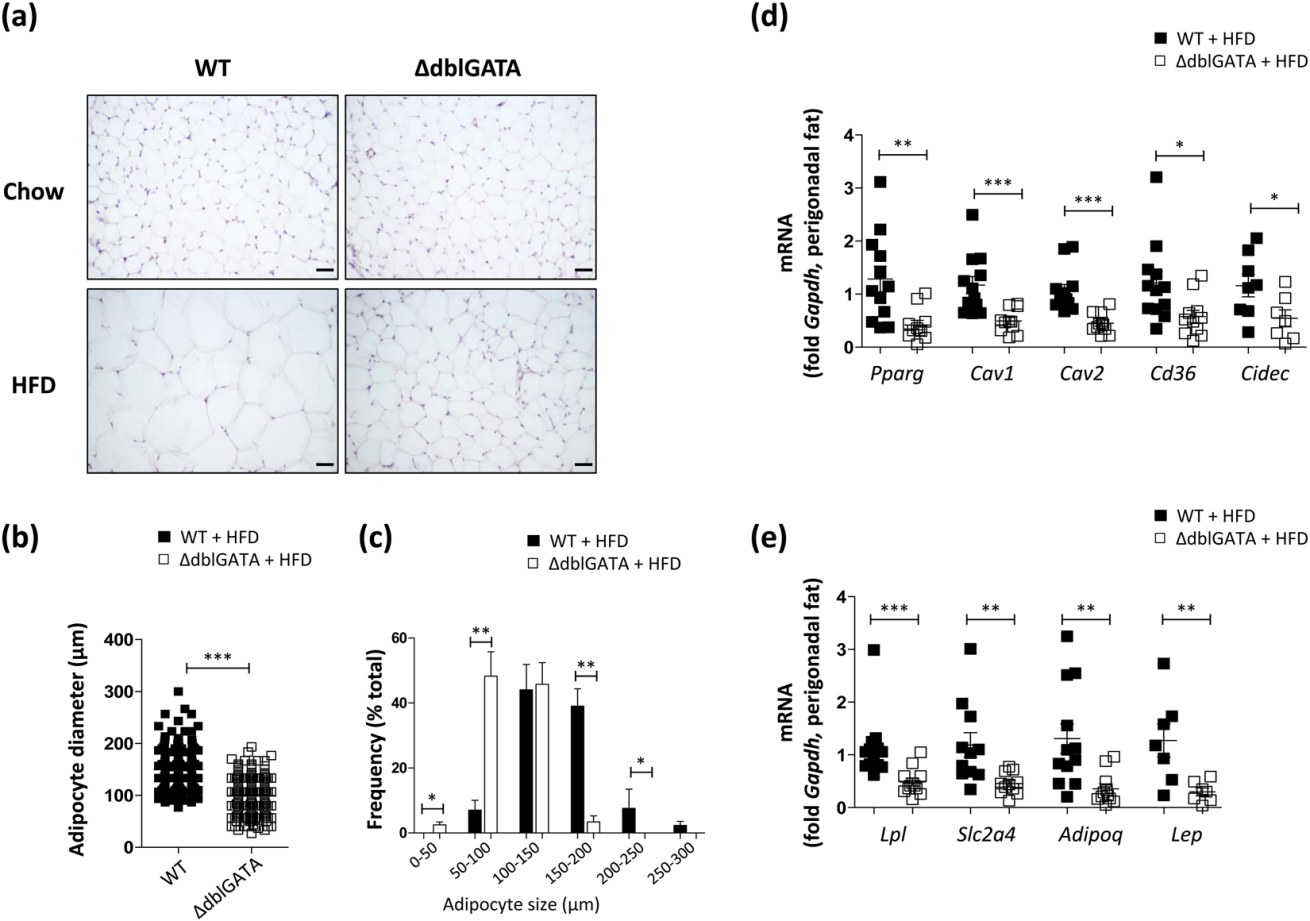
ΔdblGATA mice showed defective adipocyte enlargement and a decreased expression of genes involved in adipocyte maturation on a high-fat diet (HFD). (**a**) Haematoxylin and eosin staining of the perigonadal fat of wild-type (WT) and ΔdblGATA mice fed a HFD or chow diet. Images are representative of two independent experiments. Original magnification × 20. The scale bar represents 50 μm. (**b**,**c**) Adipocyte size and distribution. (**b**) Mean adipocyte size and (**c**) percentile distribution of adipocyte size. Three sections were examined in each group and 2–3 fields were selected from one section. *p < 0.05, **p < 0.01, ***p < 0.001 (Mann-Whitney test). (**d** and **e**) mRNA expression levels of (**d**) adipogenic and (**e**) adipokine genes in the perigonadal fat of mice on a HFD. *p < 0.05, **p < 0.01, ***p < 0.001 (Student’s *t*-test for *Pparg*, *Cav1*, *Cd36*, *Cidec*, and *Lep*, Mann-Whitney test for *Cav2*, *Lpl*, *Slc2a4*, and *Adipoq*). Graphs show the mean ± standard error of the mean.

The inability of adipose tissue to expand to accommodate excess calories causes systemic insulin resistance and hyperglycaemia^19^. As we observed an impaired WAT development in HFD-fed ΔdblGATA mice, which showed glucose intolerance, we examined whether eosinophils are required for adipocyte maturation in diet-induced obesity. A histological analysis of the perigonadal WAT of HFD-fed ΔdblGATA mice illustrated a markedly decreased adipocyte size as compared to WT mice (Fig. 3a). A quantification of the mean diameter of adipocytes in representative histological sections highlighted the significant size difference (Fig. 3b) with a significant increase in small adipocytes in HFD-fed ΔdblGATA mice (Fig. 3c). Concordantly, the expression of genes involved in adipocyte differentiation (*Pparg*) and lipid droplet formation (*Cav1*, *Cav2*, *Cd36*, and *Cidec*) was decreased in the WAT of HFD-fed ΔdblGATA mice (Fig. 3d). *Pparg* and *Cd36* expression was not decreased in the fat of 8–10 weeks-old ΔdblGATA mice fed a chow diet (Fig. S5). The expression of lipogenic markers, including *Adipoq*, which is associated with insulin sensitivity, was also decreased in the WAT of HFD-fed ΔdblGATA mice (Fig. 3e).

### Adipocytes support the migration and survival of eosinophils

**Figure 4:**
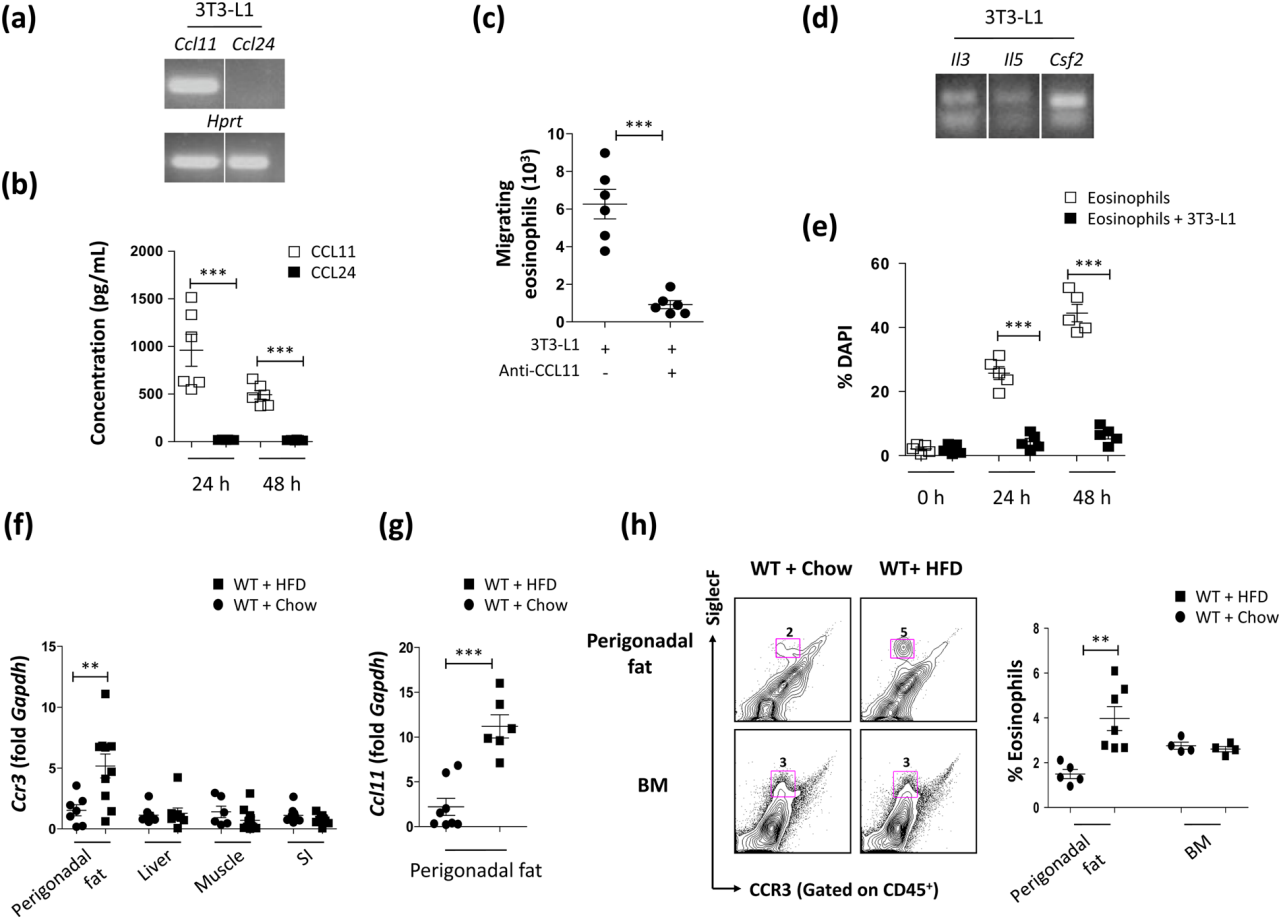
Adipocyte-derived molecules that support eosinophil migration and survival account for eosinophil infiltration in the perigonadal adipose tissue of high fat diet (HFD) fed mice. (**a**) mRNA expression of CC chemokine ligand 11 (*Ccl11*) and *Ccl24* in adipocyte-differentiated 3T3-L1 cells. Representative blots from 3 independent experiments. The full-length blots are presented in Fig. S6. ***p < 0.001 (Student’s *t*-test) (**b**) Production of CCL11 and CCL24 by cultures of adipocyte-differentiated 3T3-L1 cells. ***p < 0.001 (Student’s *t*-test) (**c**) Migration of eosinophils isolated from the peritoneal cavity of CD3-IL-5 transgenic mice toward adipocyte-differentiated 3T3-L1 cells treated with or without anti-mouse CCL11. ***p < 0.001 (Student’s *t*-test) (**d**) mRNA expression of interleukin (IL)-3 (*Il3*), IL-5 (*Il5*), and granulocyte-macrophage colony-stimulating factor (*Csf2*) in adipocyte-differentiated 3T3-L1 cells. Representative blots from 3 independent experiments. The full-length blots are presented in Fig. S6. (**e**) Viability of eosinophils isolated from the peritoneal cavity of CD3-IL-5 transgenic mice and co-cultured with or without adipocyte-differentiated 3T3-L1 cells. Non-viable eosinophils were detected by staining with 4′,6-diamidino-2-phenylindole dihydrochloride (DAPI). ***p < 0.001 (Student’s *t*-test) (**f**) mRNA expression of *Ccr3* in the perigonadal fat, liver, muscle, and small intestine (SI) of wild-type (WT) mice fed a HFD or chow. **p < 0.01 (Student’s *t*-test for fat, muscle, and SI, Mann-Whitney test for liver). (**g**) mRNA expression of *Ccl11* in the perigonadal fat of WT mice fed a HFD or chow. ***p < 0.001 (Student’s *t*-test) (**h**) Flow cytometric analysis of CCR3^+^SiglecF^+^ eosinophils in the perigonadal fat and bone marrow (BM) of WT mice fed a HFD or chow. Representative dot plots are shown. **p < 0.01 (Student’s *t*-test). Graphs show the mean ± standard error of the mean.

Since eosinophils are resident cells in the adipose tissue^20^ and ΔdblGATA mice showed an impairment of adipocyte maturation, we questioned whether adipocytes support the migration or survival of eosinophils. Eosinophils migrate in a manner dependent on CCL11 and its receptor, CC chemokine receptor (CCR) 3^2,21^. We determined that adipocyte-differentiated mouse embryonic fibroblast 3T3-L1 cells expressed *Ccl11* (Figs 4a and S6) and secreted CCL11 (Fig. 4b), which attracted eosinophils, since the application of anti-mouse CCL11 significantly inhibited eosinophil trafficking toward 3T3-L1 cells (Fig. 4c). Additionally, adipocyte-differentiated 3T3-L1 cells expressed cytokines supporting eosinophil survival, such as *Il3*, *Il5*, and *Csf2* (Figs 4d and S6). Accordingly, the viability of eosinophils was markedly increased by co-culture with adipocyte-differentiated 3T3-L1 cells (Fig. 4e). Next, we assessed eosinophil infiltration in the WAT of HFD-fed WT mice and found a significant increase in the expression of *Ccr3* and *Ccl11* in the perigonadal WAT (Fig. 4f,g). An increased frequency and number of eosinophils in the WAT was also supported by a flow cytometry analysis (Figs 4h and S7a). The frequency of eosinophils in the bone marrow was unaffected by the HFD (Fig. 4h). These results imply that eosinophils preferentially migrate to the perigonadal adipose tissue in diet-induced obesity because of the production of molecules that support their migration and survival by adipocytes.

### The perigonadal adipose tissue of eosinophil-deficient mice showed an increased inflammatory response

**Figure 5:**
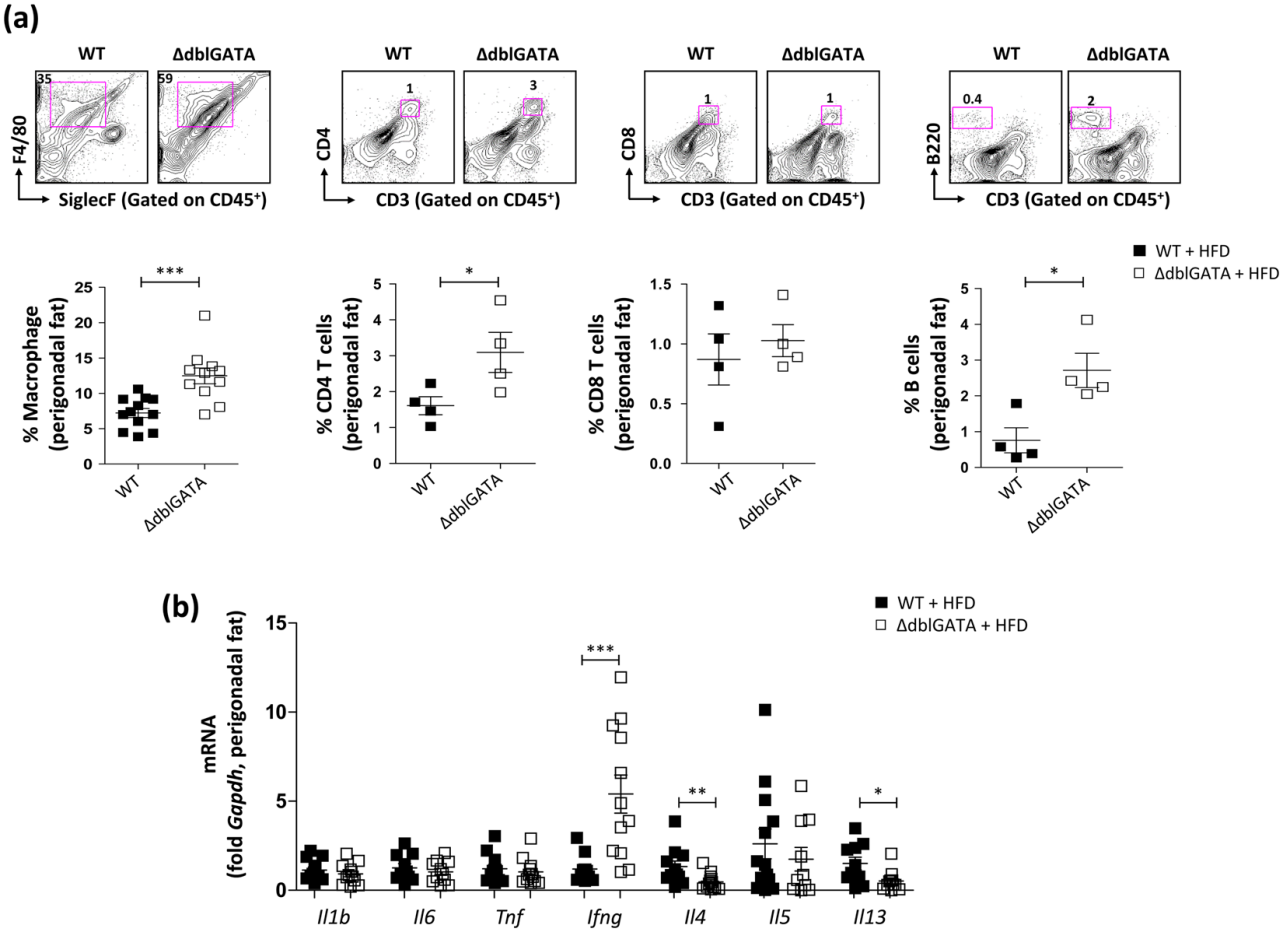
ΔdblGATA mice showed increased inflammatory responses in the perigonadal adipose tissue on a high fat diet (HFD). (**a**) Flow cytometric analysis of SiglecF^−^F4/80^+^ macrophages, CD3^+^CD4^+^ T cells, CD3^+^CD8^+^ T cells, and CD3^−^B220^+^ B cells in the perigonadal fat of wild-type (WT) and ΔdblGATA mice on a HFD. Representative dot plots are shown. *p < 0.05, ***p < 0.001 (Student’s *t*-test for macrophage, Mann-Whitney test for CD4 T cells, CD8 T cells, and B cells). (**b**) mRNA expression of innate (*Il1b*, *Il6*, and *Tnf*) and adaptive (*Ifng*, *Il4*, *Il5*, and *Il13*) immune cytokines in the perigonadal fat of mice on a HFD. *p < 0.05, **p < 0.01, ***p < 0.001 (Student’s *t*-test for *Il1b*, *Il6*, *Tnf*, and *Il4*, Mann-Whitney test for *Ifng*, *Il5*, and *Il13*). Graphs show the mean ± standard error of the mean.

Excess calorie intake results in chronic inflammation in adipose tissue involving an infiltration of various immune cells^22–26^. In obesity, the infiltrated immune cells promote the production of pro-inflammatory cytokines that inhibit adipogenesis and insulin signalling^27,28^. The total numbers of cells isolated from the perigonadal WAT of HFD-fed ΔdblGATA and WT mice were not significantly different (*p* = 0.1263, data not shown). However, significantly increased abundances of macrophages, CD4^+^ T cells, and B cells were observed in the perigonadal WAT of HFD-fed ΔdblGATA mice (Figs 5a and S7b). Although HFD-fed ΔdblGATA mice showed an increased abundance of macrophages in WAT as compared with WT mice, the ratio between the classical (M1) and alternative (M2) immune phenotypes of the macrophages was comparable between ΔdblGATA and WT mice (Fig. S8a). This finding was supported by the lack of a significant difference in the expression of *Arg1*, *Nos2*, and the ratio of *Arg1* and *Nos2* between WT and ΔdblGATA mice (Fig. S8b,c). We next measured the mRNA levels of cytokines in the perigonadal WAT and observed a marked increase in the expression of the Th1 cytokine *Ifng* in HFD-fed ΔdblGATA mice (Fig. 5b). In contrast, the expression levels of the Th2 cytokines *Il4* and *Il13* were significantly decreased in HFD-fed ΔdblGATA mice (Fig. 5b).

### The cytokine expression profile in the perigonadal adipose tissue of eosinophil-deficient mice reflected defective adipogenic maturation

**Figure 6:**
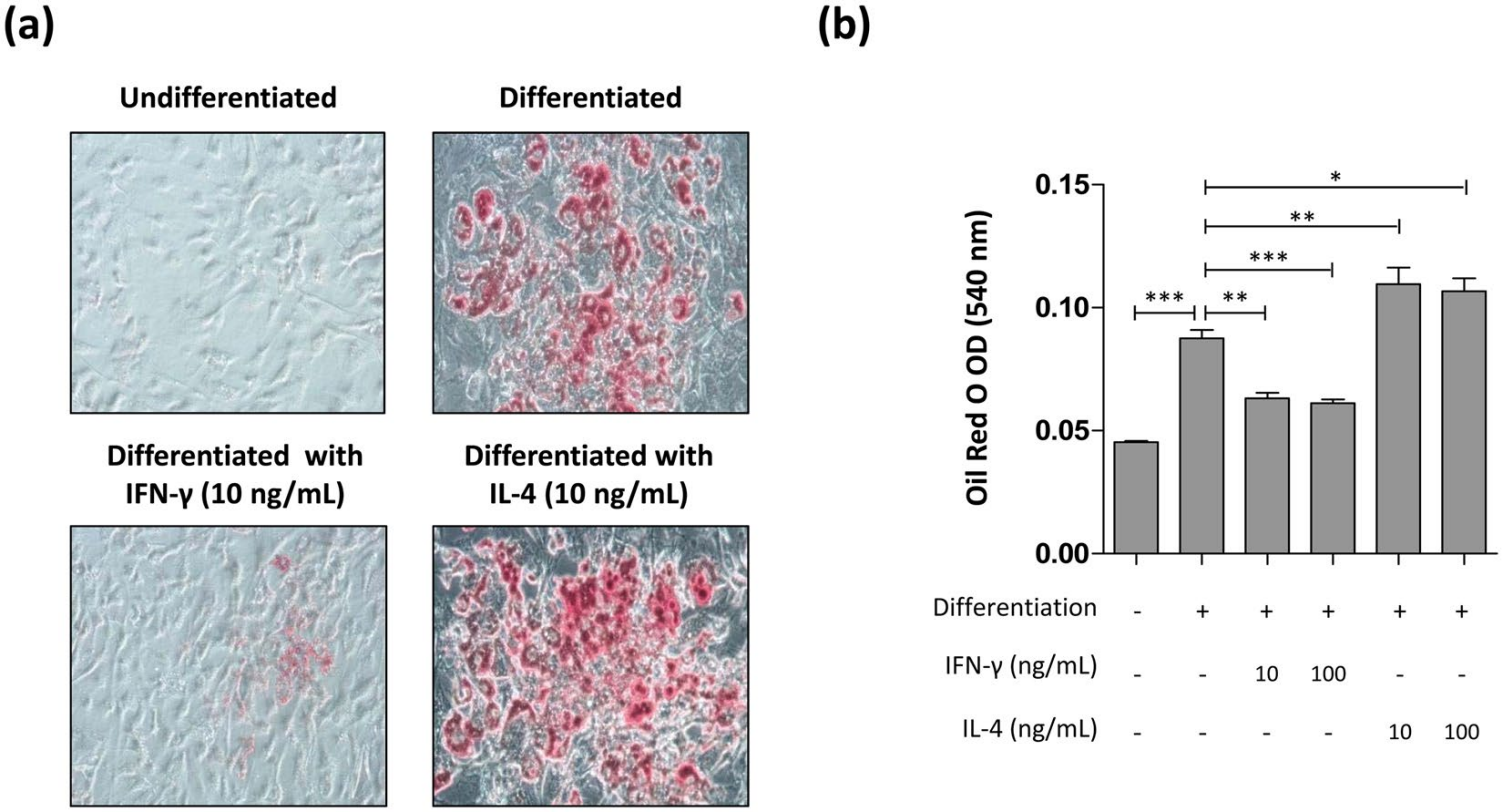
Effect of interferon (IFN)-γ and interleukin (IL)-4 treatment on 3T3-L1 adipogenesis. (**a**) 3T3-L1 cells were treated with insulin to differentiate them into adipocytes in the presence of either IFN-γ or IL-4 and stained with Oil Red O. Original magnification × 20. (**b**) Oil Red O in the adipocyte-differentiated 3T3-L1 cells was eluted using isopropanol and the optical density (OD) of the eluate was analysed. *p < 0.05, **p < 0.01, ***p < 0.001 (one-way ANOVA). Graphs show the mean ± standard error of the mean.

To determine whether the cytokine profile in the perigonadal WAT of ΔdblGATA mice affects adipocyte maturation, we induced the adipogenesis of 3T3-L1 cells in the presence of IFN-γ or IL-4. As shown in Fig. 6, adipocyte-differentiated 3T3-L1 cells treated with IFN-γ showed a significant decrease in lipid deposition as measured by Oil Red O staining. In contrast, adipocyte-differentiated 3T3-L1 cells treated with IL-4 showed an increase in lipid accumulation (Fig. 6) with significant upregulation of key adipogenic and lipogenic genes including *Cebpa*, *Acaca*, *Fasn*, and *Scd* (Fig. S9). These findings indicated that eosinophils infiltrated into the perigonadal WAT of HFD-fed mice modulate the immune microenvironment to favour adipocyte maturation.

### Eosinophil-deficient mice showed lipid accumulation in the liver

**Figure 7:**
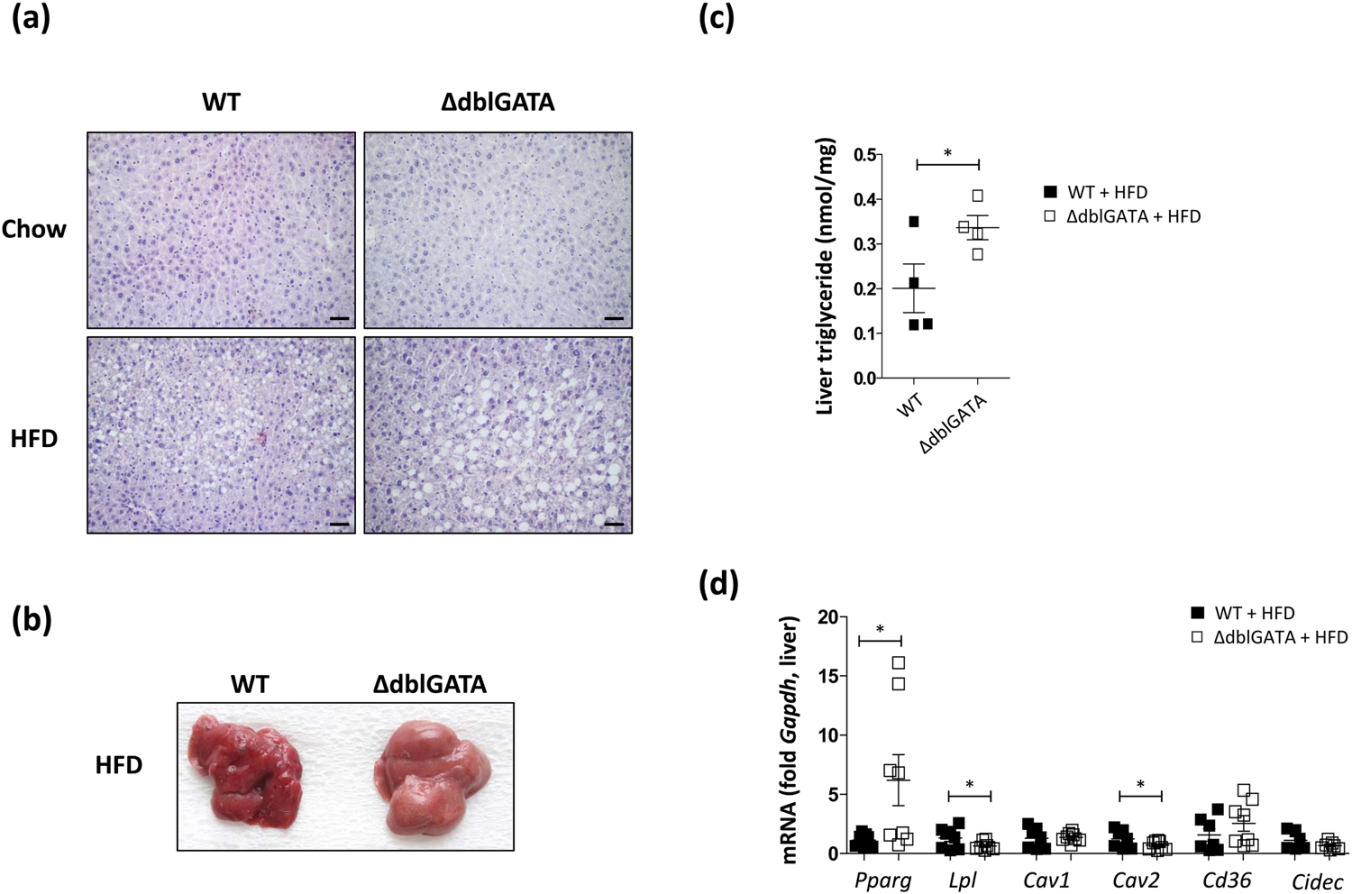
Fatty liver changes induced by high fat diet (HFD) were enhanced in ∆dblGATA mice. (**a**) Haematoxylin and eosin staining of the liver of wild-type (WT) and ΔdblGATA mice fed a HFD or chow diet. Images are representative of two independent experiments. Original magnification × 20. The scale bar represents 50 μm. (**b**) Representative image of the liver from a mouse on a HFD. (**c**) Triglyceride levels in the livers of mice fed a HFD. *p < 0.05 (Student’s *t*-test). (**d**) mRNA expression levels of adipogenic genes in the livers of mice fed a HFD. *p < 0.05 (Student’s *t*-test). Graphs show the mean ± standard error of the mean.

Excessive lipid accumulation in non-adipose tissues such as the liver, muscle, and pancreas is closely associated with insulin resistance^29^. Given that lipids tend to be stored ectopically in the absence of functional adipocytes^30^, we hypothesized that eosinophil deficiency would influence the development of ectopic fat accumulation. The livers of HFD-fed ΔdblGATA mice exhibited a paler colour and more prominent lipid deposits than those of WT mice (Fig. 7a,b). The measurement of total liver triglycerides supported the visual observation of lipid storage in the liver of HFD-fed ΔdblGATA mice (Fig. 7c). An analysis of adipogenic gene expression using real-time PCR revealed a significant increase in the expression of *Pparg* in the liver of HFD-fed ΔdblGATA mice, although the expression of *Lpl* and *Cav2* was lower in these mice (Fig. 7d). Although the lipid contents in the skeletal muscle of HFD-fed ΔdblGATA mice was comparable to in their WT counterparts, the small intestine of HFD-fed ΔdblGATA showed increased accumulation of triglycerides (Figs S10 and S11).

## Discussion

Although eosinophils have been characterized as destructive effector cells that mediate tissue damage during helminth infections and allergic diseases, they are found in various tissues under resting conditions without having obvious pro-inflammatory activities^31,32^. Accumulating evidence has indicated that tissue-resident eosinophils regulate biological processes that are not restricted to Th2 immune responses. Eosinophils in adipose tissue promote the accumulation of alternatively activated macrophages, which protect against insulin resistance induced by obesity-associated changes such as the activation of innate immune activity, alterations in fatty acid uptake, lipogenesis, and energy expenditure that can impact ectopic lipid deposition^33^. Obesity itself does not necessarily induce insulin resistance and primarily results from adipocytes expanding to buffer against excess nutrient uptake^9^. Despite recent advances in understanding the functions of eosinophils in maintaining metabolic homeostasis, their roles in adipocyte development are not fully understood. Nevertheless, multiple lines of our observation have indicated that eosinophils counteract obesity-associated metabolic dysfunctions by promoting adipocyte maturation. A significant decrease of *Ccr3* in the small intestine, lung, fat, liver, and muscle of 8–10-week-old chow diet-fed ΔdblGATA mice imply distribution of eosinophils in the metabolic organs under resting conditions (Fig. S12). In addition, a decreased expression of genes associated with lipid metabolism was observed in the small intestine (Figs 1a and S1) and perigonadal WAT (Fig. 1c) of 8–10-week-old ΔdblGATA mice, further suggesting that eosinophils regulate energy homeostasis. However, the expression of *Ccr3* significantly increased in the perigonadal WAT on a HFD (Fig. 4f), as well as the frequency and number of eosinophils (Figs 4h and S7a), implying the preferential infiltration of eosinophils into adipose tissue with obesity. Therefore, we propose that mature adipocytes release factors that support the infiltration of eosinophils into adipose tissue. Indeed, adipocyte-differentiated 3T3-L1 cells expressed CCL11, which has an eosinophil-selective chemoattractant activity^2,3^, and the CCL11 produced by 3T3-L1 cells induced the directional migration of eosinophils into adipocyte-differentiated 3T3-L1 cells (Fig. 4a–c). Adipocyte-differentiated 3T3-L1 cells also promoted the viability of eosinophils and we observed the expression of *Il3*, *Il5*, and *Csf2* (the gene encoding GM-CSF) in differentiated 3T3-L1 cells (Fig. 4d,e). These cytokines provide signals that promote the permissive proliferation and differentiation of eosinophils^2,3^. These findings contrast with previous observations that suggested an inverse correlation between the abundance of eosinophils in adipose tissue and adiposity^5,34,35^. The reasons for this difference are unclear, but mouse strains vary in their immunometabolic phenotypes^36^, as evidenced by the difference between BALB/c and C57BL/6 mice in their immune cell profiles with a HFD^37^. In line with this, chow diet-fed male C57BL/6 mice showed significantly more eosinophils in the perigonadal adipose tissue than BALB/c mice, while showing fewer eosinophils in the small intestine (Fig. S13). There are likely differences in microbiota, and thus endotoxin exposure may explain the weight difference between results of previous reports and those of our study, as exposure to microbial endotoxins may alter eosinophil maturation and activities^38^. Considering that our results are consistent with previous reports indicating eosinophils role in the regulation of metabolic homeostasis^4,5,35^, we propose that differences in mice strain and cohort might account for the divergence on eosinophil abundance in the adipose tissue between this study and previous observations.

The fat cells of adipose tissue are the only cells in the body that are designed to safely contain large amounts of fat^9^. Therefore, the impaired development of perigonadal adipose tissue observed in HFD-fed ΔdblGATA mice may lead to the accumulation of lipid outside adipose tissue. The excessive accumulation of lipids in non-adipose tissues is closely associated with insulin resistance^33^, and we propose that ectopic fat accumulation in HFD-fed ΔdblGATA mice might account for their glucose intolerance. Although increased visceral adiposity has been implicated in hepatic insulin resistance, patients with severe lipodystrophy, as well as a mouse model of lipoatrophy, manifest insulin resistance associated with lipid deposition in the liver^39,40^. The significant decrease in the expression of *Pparg*, which encodes a transcription factor involved in adipocyte differentiation, and genes associated with lipid droplet formation (*Cav1, Cav2, Cd36*, and *Cidec*) in the perigonadal WAT of HFD-fed ΔdblGATA mice (Fig. 3) suggests insufficient lipid storage in adipose tissue in the absence of eosinophils. Concordantly, the expression of *Lpl*, *Slc2a4*, and *Adipoq*, which are regulated by *Pparg* and involved in glucose homeostasis^41^, significantly decreased in the perigonadal adipose tissue of HFD-fed ΔdblGATA mice (Fig. 3). We also observed significant decreases in *Ptrf* (involved in biogenesis of caveolae), *Akt2* (involved in adipocyte differentiation and insulin signalling), and *Psmb8* (involved in expression of immunogenic epitopes), which are associated with lipodystrophies, in the WAT of HFD-fed ΔdblGATA mice (Fig. S14). We suggest that an altered immune environment in the perigonadal fat of HFD-fed ΔdblGATA accounts for the decreased expression of adipogenic genes. The exposure of preadipocytes to pro-inflammatory cytokines inhibits adipogenesis by reducing the expression of *Pparg* and inhibiting the adipogenic action of insulin^28^. Th1 cytokines, including IFN-γ, can inhibit insulin signalling and lipid droplet formation^27,42^, while Th2 cytokines, including IL-4 and IL-13, can suppress inflammatory responses in adipose tissue^43^. The increased expression of *Ifng* and decreased expression of *Il4* and *Il13* in the perigonadal fat of HFD-fed ΔdblGATA mice imply that the microenvironments of the WAT of these mice are unfavourable for adipocyte maturation. We supported this idea by demonstrating that IFN-γ attenuated lipid storage while IL-4 promoted lipid deposition in adipocyte-differentiated 3T3-L1 cells (Figs 6 and S9). These changes in cytokine expression were diet- and site-specific, since the expression of *Il4*, *Il13*, and *Ifng* was not different between WT and ΔdblGATA mice either in the perigonadal adipose tissue of chow-fed mice or in the liver of HFD-fed mice (data not shown).

Obesity is associated with an increased infiltration of macrophages, preferentially the pro-inflammatory M1 phenotype, into adipose tissue^22^, and IL-4 produced by adipose tissue eosinophils supports the polarization of anti-inflammatory M2 macrophages^5^. In agreement this with this idea, conditioned media (CM) collected from eosinophilic cell line EoL-1 cells or CM from palmitic-acid stimulated EoL-1 cells promoted M2-polarization of monocytic THP-1 cells (Fig. S15). However, the ratio between M1 and M2 macrophages was comparable between WT and ΔdblGATA mice (Fig. S8), although HFD-fed ΔdblGATA mice showed a significant decrease in the expression of *Il4* in the perigonadal WAT (Fig. 5). Given that hypoxia induces macrophage proliferation and polarization towards the M2 phenotype^44,45^, it is plausible that hypoxia determined the composition of macrophages in the adipose tissue of HFD-fed ΔdblGATA mice. The significantly elevated concentration of lactate in the perigonadal WAT of HFD-fed ΔdblGATA mice also supports this idea (Fig. S16).

Although adipose tissue is rich in stem cells that can differentiate into fat cells to contain excess energy^46^, the pro-inflammatory microenvironment of adipose tissue induced by obesity is associated with the inhibition of adipocyte maturation and increased adipocyte death^10,12^. Our data demonstrate that adipocytes provide signals to promote eosinophil migration and survival, and that eosinophils support adipocyte maturation and protect adipose tissue against inflammatory changes. Based on our findings, we propose that eosinophils and adipocytes bidirectionally cooperate to promote metabolic homeostasis in diet-induced obesity.

## Methods

### Mice

BALB/c WT mice (Orientbio, Gyeonggi, Korea) and ∆dblGATA mice (C.129S1(B6)-Gata1^tm6Sho^/J, Jackson Laboratory, Bar Harbor, ME, USA) were housed under standard temperature and humidity in the specific pathogen-free facilities of Gachon University. Cluster of differentiation (CD) 3-IL-5 transgenic mice (NJ.1638, *Il5*^*Tg*^) were kindly provided by Dr. Jamie Lee (Mayo Clinic, Scottsdale, AZ, USA) and housed in the specific pathogen-free facilities of Tel Aviv University. Animal procedures were reviewed and approved by the Center of Animal Care and Use of Lee Gil Ya Cancer and Diabetes Institute, Gachon University (Number: LCDI-2016-0060) or by the Animal Care Committee of Tel-Aviv University (Number: M-13-029, M-13–30), and were performed in accordance with its regulations and guidelines regarding the care and use of animals for experimental procedures.

### RNA sequencing and bioinformatics analysis

RNA isolated from the small intestine of 8–10-week-old WT (n = 4) and ∆dblGATA mice (n = 4) was subjected to RNA sequencing at the Cincinnati Children’s Hospital Medical Center sequencing core. Sequencing data were demultiplexed and reads were mapped to the mm10 mouse genome reference using TopHat^47^. The total number of mapped reads per transcript was determined and the data were normalized to detect the number of fragments per kilobase of exon per million fragments mapped (FPKM) using Cufflinks^48^. An FPKM >0.1 in at least 1 of the analysed samples was used to filter for potentially significant gene expression. Transcripts with fold-change values >2 with a false discovery rate-corrected *p*-value < 0.05 were included as differentially expressed genes. Functional groups and pathways encompassing the differentially expressed genes were identified based on a Gene Ontology (GO) analysis using the Database for Annotation, Visualization, and Integrated Discovery. GO terms and pathways having an enrichment score >1.3, *p*-value < 0.05, and number of genes ≥3 were defined as significantly enriched^49^.

### HFD feeding and metabolic studies

Eight-to-ten-week-old male mice were fed a HFD (60% fat, D12492, Research Diets, New Brunswick, NJ, USA) or a chow diet (5.0% fat, 5053, LabDiet, St. Louis, MO, USA) for 12 weeks. Body weight was measured weekly. Fasting insulin concentrations were measured by enzyme-linked immunosorbent assay (ELISA, Shibayagi, Gunma, Japan). For glucose tolerance tests (GTTs), glucose (1.5 g/kg) was injected intraperitoneally after starvation for 16 h and blood glucose was measured using a glucometer (Allmedicus, Kyunggi, Korea). For *in vivo* insulin signalling analysis, mice were anesthetized after overnight fasting. Insulin (0.75 U/kg) or saline was infused into the liver via the portal vein. Five minutes after infusion, liver, perigonadal fat, and skeletal muscle were quickly excised and snap-frozen in liquid nitrogen^50^. At sacrifice, all mice were weighed and the livers and perigonadal fat were removed and weighed.

### Total protein extraction and western blot analysis

The tissue lysates were prepared in ice-cold tissue lysis buffer (50 mM Tris-HCl, pH 7.5; 150 mM NaCl; 1% Nonidet P-40; 0.5% sodium deoxycholate; 0.1% SDS) containing 50 mM NaF, 2 mM Na_3_VO_4_, protease inhibitors (Sigma-Aldrich, St. Louis, MO, USA) and phosphatase inhibitors (Sigma-Aldrich) and total protein was extracted as previously described^50^. Samples from tissue lysates were resolved by SDS-PAGE and then transferred to a nitrocellulose membrane. After 1 h blocking at 4 °C using 5% BSA in phosphate-buffered saline containing 0.1% Tween-20 (PBST), the membrane was incubated overnight with antibodies against phospho-IRβ (sc-25103, Santa Cruz Biotechnology, Santa Cruz, CA, USA), phospho-Akt (SAB5600064, Sigma-Aldrich), and α-tubulin (T9026, Sigma-Aldrich) in PBST at 4 °C. After 3 PBST washes, membranes were incubated with secondary antibody for 1 h at room temperature. Chemiluminescence was performed using a SuperSignal West Pico Chemiluminescent Substrate (Thermo Fisher Scientific, Waltham, MA, USA).

### PCR analysis

Total RNA was extracted using QIAzol^®^ lysis reagent (Qiagen, Hilden, Germany) and subsequently column-purified with an RNeasy^®^ Mini Kit (Qiagen). RNA (500 ng) was treated with DNase I (New England Biolabs, Ipswich, MA, USA) and cDNA was synthesized using an iScript™ cDNA synthesis kit (Bio-Rad Laboratories, Hercules, CA, USA). Real-time PCR was performed using iQ SYBR^®^ Green Supermix (Bio-Rad Laboratories) on a CFX Connect™ real-time PCR detection system (Bio-Rad Laboratories). Reverse-transcription PCR was performed using Bio-ReadyMix (Bio-Lab, Jerusalem, Israel) and the amplified DNA products were electrophoresed on 2% agarose gels. The primers are detailed in Supplementary Tables S4 and S5.

### Histology

The perigonadal fat and liver specimens were fixed in 10% buffered formalin and embedded in paraffin. Multiple 4-μm sections were stained with haematoxylin and eosin and visualized using a CX41 microscope (Olympus, Tokyo, Japan).

### Preparation of cell suspension

Perigonadal adipose tissues were incubated with 0.2% collagenase II (Sigma-Aldrich) in Roswell Park Memorial Institute (RPMI) 1640 medium/0.5% BSA with continuous stirring at 37 °C for 30 min. Bone marrow cells were collected by flushing the femurs with RPMI 1640 medium/5% FBS. Isolated cells were filtered through a 100-μM cell strainer and red blood cells were lysed. For the enrichment of leukocytes, the cells were subjected to density-gradient centrifugation in 40%/75% Percoll^®^ (Sigma-Aldrich). The cells harvested from the interface were washed and used in subsequent assays.

### Flow cytometry

To characterize the cell surface phenotype, isolated cells were resuspended in PBS containing 10% FCS, 20 mM HEPES, and 10 mM EDTA. After blocking Fc receptors with anti-mouse CD16/CD32 (2.4G2, BD Biosciences, San Diego, CA, USA) for 15 min at 4 °C, the cells were stained for 30 min at 4 °C with the following antibodies: mAb against CCR3 (83101) from R&D Systems (Minneapolis, MN, USA); anti-SiglecF (E50-2440) from BD Biosciences; anti-CD45 (30-F11), F4/80 (BM8), CD3 (145-2C11), and B220 (RA3-6B2) from BioLegend (San Diego, CA, USA); and anti-CD4 (RM4-5) and CD8 (53–6.7) from eBioscience (San Diego, CA, USA). Each sample was analysed with a FACSCalibur™ flow cytometer (BD Biosciences) and the data were processed using FlowJo software (Tree Star, Ashland, OR, USA).

### Isolation of eosinophils

Eosinophils were isolated from the peritoneal cavity of CD3-IL-5 transgenic mice as described^20^ and used for chemotaxis assay and co-culture with adipocyte-differentiated 3T3-L1 cells. Total cells were extracted and subjected to lymphocyte depletion using a MACS^®^ system with antibodies against CD90.2 and CD45R (Miltenyi Biotec, Auburn, CA, USA). The purity and viability of the isolated cells were determined by flow cytometry and were consistently >95%.

### Adipogenesis of 3T3-L1 cells

3T3-L1 preadipocytes were cultured in Dulbecco’s Modified Eagle’s medium (DMEM) containing 10% FBS, 10 mg/mL streptomycin, and 100 U/mL penicillin at 37 °C in 5% CO_2_. To induce adipocyte differentiation, at 2 days post-confluence, the cells were treated with 0.5 mM isobutylmethylxanthine (Sigma-Aldrich), 10 μM dexamethasone (Sigma-Aldrich), and 1 μg/mL insulin (Sigma-Aldrich) for 72 h, followed by maintenance in DMEM containing 10% FBS and 1 μg/mL insulin. To examine the effects of cytokines on adipogenesis, the 3T3-L1 cells were treated with IFN-γ (PeproTech, Rocky Hill, NJ, USA) or IL-4 (PeproTech) during their differentiation into adipocytes.

### ELISA

The concentrations of CCL11 (eotaxin-1) and CCL24 (eotaxin-2) secreted from adipocyte-differentiated 3T3-L1 cells were measured by ELISA according to the manufacturer’s instructions (R&D Systems).

### Chemotaxis assay

Chemotaxis was assayed using Transwell^®^ inserts (Corning, Corning, NY, USA) with a 3-μm pore diameter. Differentiated 3T3-L1 cells were grown in DMEM containing 10% FBS at 450 μL per well until they achieved confluence. Eosinophils were loaded into each upper insert (200,000 cells in 450 μL medium). To examine the effect of chemokine blockade, anti-mouse CCL11 (Peprotech, Rehovot, Israel) was added (50 μg/mL) to the lower wells. After 24 h, the inserts were removed and the migrated cells were stained and enumerated by flow cytometry.

### Cell viability assay

Eosinophils were cultured for 24 h with or without adipocyte-differentiated 3T3-L1 cells. Non-viability was determined by staining cells with 4′,6-diamidino-2-phenylindole dihydrochloride (Sigma-Aldrich) and analysing them by flow cytometry.

### Oil Red O staining

To evaluate lipid accumulation, adipocyte-differentiated 3T3-L1 cells were stained with Oil Red O (Sigma-Aldrich). For quantification, the dye was eluted by adding 100% isopropanol and the absorbance of the eluate was measured at 540 nm.

### Liver triglyceride quantification

Liver triglycerides were determined with an assay kit according to the manufacturer’s instructions (Biovision, Palo Alto, CA, USA).

### Statistical analysis

Two-group comparisons were performed by Student’s *t*-test or Mann-Whitney test. Data differences between groups were examined for statistical significance using two-way ANOVA with the Bonferroni post hoc test or one-way ANOVA with the Tukey post hoc test. A *p*-value < 0.05 was considered significant. The data are presented as the mean ± standard error of the mean. GraphPad Prism 5 (GraphPad, San Diego, CA, USA) was used for data analysis.

## Electronic supplementary material

Supplementary info
