# Supplementary material for: Eosinophils support adipocyte maturation and promote glucose tolerance in obesity
Source: Sci Rep. 2018 Jul 2;8:9894. doi: 10.1038/s41598-018-28371-4 (PMC6028436; doi:10.1038/s41598-018-28371-4)
Supplement: Supplementary file 1 — Supplementary info [file 41598_2018_28371_MOESM1_ESM.docx]

**Eosinophils support adipocyte maturation and promote glucose tolerance in obesity**

Eun-Hui Lee^1,†^, Michal Itan^2,†^, Jinsun Jang^1,3^, Hyeon-Jung Gu^1^, Perri Rozenberg^2^, Melissa K. Mingler^4^, Ting Wen^4^, Jiyoung Yoon^1^, Shi-Young Park^5,6^, Joo Young Roh^3^, Cheol Soo Choi^5,6^, Woo-Jae Park^7,8^, Ariel Munitz^2^, YunJae Jung^1,8,*^

^1^Department of Microbiology, School of Medicine, Gachon University, Incheon, 21999, Korea; ^2^Department of Clinical Microbiology and Immunology, The Sackler School of Medicine, Tel-Aviv University, Ramat Aviv, 69978, Israel; ^3^Department of Dermatology, Gachon University of Gil Medical Center, Incheon, 21565, Korea; ^4^Division of Allergy and Immunology, Cincinnati Children’s Hospital Medical Center, University of Cincinnati College of Medicine, Cincinnati, OH, 45229, USA; ^5^Department of Molecular Medicine, Lee Gil Ya Cancer and Diabetes Institute, School of Medicine, Gachon University, Incheon, 21999, Korea; ^6^Korea Mouse Metabolic Phenotyping Center, Lee Gil Ya Cancer and Diabetes Institute, School of Medicine, Gachon University, Incheon, 21999, Korea; ^7^Department of Biochemistry, School of Medicine, Gachon University, Incheon, 21999, Korea; ^8^Gachon Advanced Institute for Health Science & Technology, Gachon University, Incheon, 21999, Korea

^†^These authors contributed equally to this work.

^*^ Corresponding author

**Supplementary figure legends**

**Figure S1. Real-time PCR analysis of the top 10 downregulated genes in the small intestine (SI) of 8–10-week-old ∆dblGATA mice fed a chow diet.** WT indicates wild-type mice. **p* < 0.05, ****p* < 0.001 (Student’s *t*-test for *Cepbe*, *Faim*, *Drd2*, and *Thsd1*, Mann-Whitney test for *Retnlg*, *Ccr3*, *Alox15*, *Serpinb2*, *Slco4c1*, and *Nlrp12*). Graphs show the mean ± standard error of the mean.

**Figure S2. Functional interactions of differentially expressed genes between small intestine of wild-type and ΔdblGATA mice fed a high-fat diet.** The enriched pathways were predicted using the ClueGO (<http://apps.cytoscape.org/apps/cluego>) plugin of Cytoscape.

**Figure S3. Analysis of parameters related to energy homeostasis mice fed a high-fat diet (HFD) or chow.** (a) Weight of perigonadal fat and liver of mice on an HFD or chow diet. ****p* < 0.001 (Student’s *t*-test). (b) Insulin tolerance test of the indicated mice fasted for 4 h (*n* = 10–13 mice/group). **p* < 0.05, (Student’s *t*-test), WT vs. ΔdblGATA mice. (c) Fasting blood insulin levels on an HFD or chow diet. *** *p* < 0.001 (Student’s *t*-test). Graphs show the mean ± standard error of the mean.

**Figure S4. Analysis of lipid in serum and stool of mice fed a high fat diet (HFD) or chow.** (a) Triglyceride and free fatty acid (FFA) levels in serum were measured with assay kits using the instructions provided by the manufacturer (Biovision, Palo Alto, CA, USA). Graphs show the mean ± SEM values. ***P* < 0.01 (Student’s *t*-test). (b) Analysis of faecal lipids by thin layered chromatography (TLC). Lipids were extracted using chloroform/methanol and separated by TLC using heptane/isopropyl ether/acetic acid as the developing solvent. Lipids were visualized by charring the plate with copper sulfate and heating to 100°C. (c) Energy intake and energy expenditure of wild-type (WT) and ΔdblGATA mice were measured (2 days of acclimation followed by 24 h of measurement) using a Comprehensive Laboratory Animal Monitoring System (Columbus Instruments, Columbus, OH, USA) after 8 weeks on an HFD.

**Figure S5. mRNA expression levels of adipogenic genes in the perigonadal white adipose tissue (WAT) of 8–10-week-old mice on a chow diet.** mRNA expression of *Pparg*, *Cav1*, *Cav2*, *Cd36*, and *Cidec* in the WAT of wild-type (WT) and ΔdblGATA mice. **p* < 0.05 (Student’s *t*-test for *Pparg*, Mann-Whitney test for *Cav1*, *Cav2*, *Cd36*, and *Cidec*). Graphs show the mean ± standard error of the mean.

**Figure S6. mRNA expression of *Ccl11, Ccl24, Il3, Il5,* and *Csf2* in adipocyte-differentiated 3T3-L1 cells**. Lane M, size marker; lane 1–3, adipocyte-differentiated 3T3-L1 cells; lane N, no template control.

**Figure S7. Total number of cells per gram of perigonadal fat.** (a) Number of eosinophils and (b) macrophages **p* < 0.05, ***p* < 0.01, (Student’s *t*-test).

**Figure S8. Proportions of M1 and M2 macrophages in the perigonadal adipose tissue of mice on a high fat diet (HFD).** (a) Flow cytometric analysis of M1 and M2 macrophages in the perigonadal fat of wild-type (WT) and ΔdblGATA mice on a HFD. M1 and M2 indicates CD45^+^F4/80^+^CD11c^+^CD206^-^ and CD45^+^F4/80^+^CD11c^-^CD206^+^ macrophages, respectively. Anti-CD11c (HL3) was purchased from BD Biosciences (San Diego, CA, USA) and CD206 (C068C2) was purchased from BioLegend (San Diego, CA, USA). Representative dot plots are shown. (b) mRNA expression of *Arg1* and *Nos2* in the perigonadal fat of mice on a HFD. (c) Ratio of *Arg1*/*Nos2* expression in the perigonadal fat of mice on a HFD. Graphs show the mean ± standard error of the mean.

**Figure S9. mRNA expression levels of adipogenic and lipogenic genes in adipocyte-differentiated 3T3-L1 cells.** mRNA expression of (a) adipogenic genes (*Pparg*, *Cebpea*, and *Lpl*) and (b) lipogenic genes (*Acaca*, *Fasn*, and *Scd*) in adipocyte-differentiated 3T3-L1 cells treated with IFN-γ or IL-4. **p* < 0.05, ***p* < 0.01, ****p* < 0.001 (Student’s *t*-test). Graphs show the mean ± standard error of the mean.

**Figure S10. Analysis of tissue lipids by thin-layer chromatography (TLC).** Lipids were extracted from the small intestine and skeletal muscle of the indicated mice using chloroform/methanol and separated by TLC using heptane/isopropyl ether/acetic acid as the developing solvent. Lipids were visualized by charring the plate with copper sulfate and heating to 100°C.

**Figure S11. Full-length thin-layer chromatography (TLC) blot**. Full-length TLC of the small intestine and skeletal muscle of the indicated mice.

**Figure S12. Real-time PCR analysis of *Ccr3* in various organs of 8–10-week-old mice on a chow diet.** mRNA expression of *Ccr3* in the small intestine (SI), lung, fat, liver, and muscle of WT and ΔdblGATA mice. ***p* < 0.01, ****p* < 0.001 (Student’s *t*-test). Graphs show the mean ± standard error of the mean.

**Figure S13. Proportion of eosinophils in the perigonadal adipose tissue and small intestine (SI) of BALB/c and C57BL/6 mice on a chow diet.** Flow cytometric analysis of CCR3^+^SiglecF^+^ eosinophils in the perigonadal fat and SI of BALB/c and C57BL/6 mice. Representative dot plots are shown. **p* < 0.05 (Mann-Whitney test). Graphs show the mean ± standard error of the mean.

**Figure S14. Real-time PCR analysis of lipodystrophy-associated genes in the perigonadal fat of mice fed a high-fat diet (HFD).** WT indicates wild-type mice. Graphs show the mean ± standard error of the mean. **p* < 0.05, ***p* < 0.01, ****p* < 0.001 (Student’s *t*-test for *Pparg*, *Cav1*, *Ptrf*, *Bscl2*, *Lmna*, *Zmpste24*, *Akit2*, *Psmb8*, and *Plin1*, Mann-Whitney test for *Cidec* and *Agpat2*).

**Figure S15. Real-time PCR analysis of M1 and M2 macrophages-related markers in THP-1 cells.** (a) mRNA expression of M1-related and (b) M2-related genes found in THP-1 cells harvested from control and all different polarizing treatments. Control: no stimulation; M0: phorbol 12-myristate 13-acetate (PMA)-only; M1: PMA + lipopolysaccharide + IFN-γ; M2: PMA + IL-4; M0 + EoL-1 CM: M0 cells treated with conditioned media (CM) collected from eosinophilic cell line EoL-1; M0 + EoL-1 PACM: M0 cells treated with CM collected from palmitic acid (PA)-stimulated EoL-1 cells. **p* < 0.05, ***p* < 0.01, ****p* < 0.001 (Student’s *t*-test). Graphs show the mean ± standard error of the mean.

**Figure S16. Lactate concentrations in the white adipose tissue (WAT) of mice on a high fat diet (HFD).** Lactate concentrations in the WAT of wild-type (WT) and ΔdblGATA were determined using an assay kit following the instructions provided by the manufacturer (Eton Bioscience, San Diego, CA, USA). **p* < 0.05 (Mann-Whitney test). Graphs show the mean ± standard error of the mean.

**Table S1. List of downregulated genes in the small intestine of ΔdblGATA mice as determined by RNA-sequencing.** FDR indicates the false discovery rate.

| **GenBank accession no.** | **Symbol** | **Description** | **Fold change** | **FDR corrected *p*-value** |
| --- | --- | --- | --- | --- |
| NM_181596 | *Retnlg* | resistin like gamma | 1.8E-03 | 1.8E-02 |
| NM_009914 | *Ccr3* | chemokine (C-C motif) receptor 3 | 8.4E-03 | 2.8E-02 |
| NM_207131 | *Cebpe* | CCAAT/enhancer binding protein (C/EBP), epsilon | 1.0E-02 | 3.2E-02 |
| NR_045050 | *9130015A21Rik* | RIKEN cDNA 9130015A21 gene | 1.2E-02 | 1.9E-02 |
| NM_009660 | *Alox15* | arachidonate 15-lipoxygenase | 1.3E-02 | 2.5E-02 |
| NM_001174170 | *Serpinb2* | serine (or cysteine) peptidase inhibitor, clade B, member 2 | 1.7E-02 | 2.9E-02 |
| NM_001177896 | *9830107B12Rik* | RIKEN cDNA 9830107B12 gene | 1.8E-02 | 1.9E-02 |
| NM_172658 | *Slco4c1* | solute carrier organic anion transporter family, member 4C1 | 2.3E-02 | 3.1E-02 |
| NM_001122851 | *Faim* | Fas apoptotic inhibitory molecule | 2.9E-02 | 4.3E-02 |
| NM_001033431 | *Nlrp12* | NLR family, pyrin domain containing 12 | 3.3E-02 | 3.1E-02 |
| NM_010077 | *Drd2* | dopamine receptor D2 | 3.7E-02 | 4.1E-02 |
| NR_045075 | *A330102I10Rik* | - | 3.8E-02 | 3.8E-02 |
| NM_001205253 | *Thsd1* | thrombospondin, type I, domain 1 | 3.9E-02 | 3.3E-02 |
| NM_001033253 | *Plekhg1* | pleckstrin homology domain containing, family G  (with RhoGef domain) member 1 | 4.9E-02 | 1.1E-02 |
| NM_145423 | *Slc5a8* | solute carrier family 5 (iodide transporter), member 8 | 5.1E-02 | 4.8E-02 |
| NM_008089 | *Gata1* | GATA binding protein 1 | 5.5E-02 | 3.1E-02 |
| NR_045866 | *Gm6116* | predicted gene 6116 | 5.7E-02 | 3.8E-02 |
| NM_009962 | *Gpr44* | - | 5.8E-02 | 3.8E-02 |
| NM_001252413 | *Rpain* | RPA interacting protein | 6.2E-02 | 4.9E-02 |
| NM_198416 | *Zzz3* | zinc finger, ZZ domain containing 3 | 6.3E-02 | 3.3E-02 |
| NM_018857 | *Msln* | mesothelin | 6.9E-02 | 3.7E-02 |
| NM_001286424 | *Fut10* | fucosyltransferase 10 | 7.0E-02 | 4.8E-02 |
| NM_001202445 | *Mapk8ip1* | mitogen-activated protein kinase 8 interacting protein 1 | 7.4E-02 | 4.9E-02 |
| NM_026820 | *Ifitm1* | interferon induced transmembrane protein 1 | 7.5E-02 | 1.7E-02 |
| NM_001134459 | *Prickle2* | prickle homolog 2 (Drosophila) | 7.7E-02 | 3.3E-02 |
| NM_001282072 | *Deaf1* | deformed epidermal autoregulatory factor 1 (Drosophila) | 7.9E-02 | 3.3E-02 |
| NM_008370 | *Il5ra* | interleukin 5 receptor, alpha | 8.2E-02 | 2.8E-02 |
| NM_001033339 | *Mmp25* | matrix metallopeptidase 25 | 8.3E-02 | 2.3E-02 |
| NM_001039701 | *Il1rn* | interleukin 1 receptor antagonist | 8.6E-02 | 2.6E-02 |
| NM_177303 | *Lrrn4* | leucine rich repeat neuronal 4 | 9.6E-02 | 4.2E-02 |
| NM_016885 | *Emcn* | endomucin | 9.8E-02 | 3.3E-02 |
| NM_010050 | *Dio2* | deiodinase, iodothyronine, type II | 9.8E-02 | 2.6E-02 |
| NM_001285877 | *Pafah2* | platelet-activating factor acetylhydrolase 2 | 9.9E-02 | 2.6E-02 |
| NM_001252450 | *Cldn25* | claudin 25 | 1.1E-01 | 1.1E-02 |
| NM_001163468 | *Phtf1* | putative homeodomain transcription factor 1 | 1.2E-01 | 3.5E-02 |
| NM_001111022 | *Runx1* | runt related transcription factor 1 | 1.2E-01 | 4.5E-02 |
| NM_013580 | *Ldhc* | lactate dehydrogenase C | 1.2E-01 | 3.4E-02 |
| NM_025869 | *Dusp26* | dual specificity phosphatase 26 (putative) | 1.2E-01 | 2.5E-02 |
| NM_033610 | *Sncb* | synuclein, beta | 1.3E-01 | 3.8E-02 |
| NM_001285816 | *Tmem53* | transmembrane protein 53 | 1.3E-01 | 3.3E-02 |
| NM_001164708 | *Hspb2* | heat shock protein 2 | 1.3E-01 | 4.5E-02 |
| NM_024444 | *Cyp4f18* | cytochrome P450, family 4, subfamily f, polypeptide 18 | 1.3E-01 | 2.6E-02 |
| NM_001285433 | *Eef1d* | eukaryotic translation elongation factor 1 delta  (guanine nucleotide exchange protein) | 1.4E-01 | 3.2E-02 |
| NM_001002786 | *Rab44* | RAB44, member RAS oncogene family | 1.4E-01 | 2.6E-02 |
| NR_045753 | *Gm16596* | - | 1.4E-01 | 1.2E-02 |
| NM_175309 | *Upk3b* | uroplakin 3B | 1.5E-01 | 4.9E-02 |
| NM_007946 | *Epx* | eosinophil peroxidase | 1.5E-01 | 4.6E-02 |
| NM_017379 | *Tuba8* | tubulin, alpha 8 | 1.5E-01 | 2.6E-02 |
| NM_130858 | *Nxph3* | neurexophilin 3 | 1.5E-01 | 3.3E-02 |
| NM_001044704 | *Zscan21* | zinc finger and SCAN domain containing 21 | 1.5E-01 | 1.1E-02 |
| NM_177083 | *B430306N03Rik* | RIKEN cDNA B430306N03 gene | 1.6E-01 | 3.0E-03 |
| NM_145437 | *Cd300ld* | CD300 molecule-like family member d | 1.6E-01 | 2.4E-02 |
| NM_001163591 | *Stx11* | syntaxin 11 | 1.6E-01 | 3.8E-02 |
| NM_007718 | *Ccr1l1* | chemokine (C-C motif) receptor 1-like 1 | 1.6E-01 | 3.3E-02 |
| NM_008311 | *Htr2b* | 5-hydroxytryptamine (serotonin) receptor 2B | 1.6E-01 | 3.0E-02 |
| NM_178165 | *Fcrl1* | Fc receptor-like 1 | 1.6E-01 | 2.9E-02 |
| NM_178796 | *A530064D06Rik* | RIKEN cDNA A530064D06 gene | 1.6E-01 | 1.4E-02 |
| NR_040426 | *Rbakdn* | Rbak downstream neighbor (non-protein coding) | 1.7E-01 | 3.6E-02 |
| NM_001005860 | *Clec4a4* | C-type lectin domain family 4, member a4 | 1.7E-01 | 2.8E-02 |
| NM_183312 | *Synm* | synemin, intermediate filament protein | 1.7E-01 | 2.8E-02 |
| NM_001271001 | *Gfra4* | glial cell line derived neurotrophic factor family receptor alpha 4 | 1.7E-01 | 3.1E-02 |
| NM_198630 | *1810024B03Rik* | RIKEN cDNA 1810024B03 gene | 1.8E-01 | 4.4E-02 |
| NM_175012 | *Grp* | gastrin releasing peptide | 1.9E-01 | 3.5E-02 |
| NM_008344 | *Igfbp6* | insulin-like growth factor binding protein 6 | 1.9E-01 | 4.7E-02 |
| NM_001146710 | *Ppp1r18* | protein phosphatase 1, regulatory subunit 18 | 2.0E-01 | 4.8E-02 |
| NR_045949 | *Gm16325* | - | 2.0E-01 | 4.3E-02 |
| NM_001276676 | *Syt6* | synaptotagmin VI | 2.0E-01 | 4.7E-02 |
| NM_178611 | *Lair1* | leukocyte-associated Ig-like receptor 1 | 2.0E-01 | 3.1E-02 |
| NM_011990 | *Slc7a11* | solute carrier family 7  (cationic amino acid transporter, y+ system), member 11 | 2.1E-01 | 1.7E-02 |
| NM_145703 | *Kcnip2* | Kv channel-interacting protein 2 | 2.1E-01 | 3.1E-02 |
| NM_001243046 | *Dlg2* | discs, large homolog 2 (Drosophila) | 2.1E-01 | 3.4E-02 |
| NM_001033405 | *Treml2* | triggering receptor expressed on myeloid cells-like 2 | 2.1E-01 | 2.6E-02 |
| NM_001285854 | *Matk* | megakaryocyte-associated tyrosine kinase | 2.2E-01 | 2.8E-02 |
| NM_001005748 | *Phactr1* | phosphatase and actin regulator 1 | 2.2E-01 | 2.8E-02 |
| NM_211357 | *Eya3* | eyes absent 3 homolog (Drosophila) | 2.2E-01 | 3.3E-02 |
| NM_001169153 | *Cd300lf* | CD300 antigen like family member F | 2.2E-01 | 2.8E-02 |
| NM_001271497 | *Il15ra* | interleukin 15 receptor, alpha chain | 2.2E-01 | 1.1E-02 |
| NM_001110300 | *Ap1m2* | adaptor protein complex AP-1, mu 2 subunit | 2.2E-01 | 3.3E-02 |
| NM_053116 | *Wnt16* | wingless-related MMTV integration site 16 | 2.2E-01 | 1.7E-02 |
| NM_026571 | *Lhfpl5* | lipoma HMGIC fusion partner-like 5 | 2.3E-01 | 2.7E-02 |
| NM_001077362 | *Fhl1* | four and a half LIM domains 1 | 2.3E-01 | 2.6E-02 |
| NM_007675 | *Ceacam10* | carcinoembryonic antigen-related cell adhesion molecule 10 | 2.3E-01 | 2.6E-02 |
| NM_001037809 | *Cdh3* | cadherin 3 | 2.4E-01 | 4.3E-02 |
| NM_001110009 | *Apoc1* | apolipoprotein C-I | 2.4E-01 | 3.3E-02 |
| NM_001080388 | *Mark2* | MAP/microtubule affinity-regulating kinase 2 | 2.4E-01 | 4.1E-02 |
| NR_038028 | *B230208H11Rik* | - | 2.4E-01 | 2.6E-02 |
| NM_001252472 | *Cd84* | CD84 antigen | 2.5E-01 | 1.1E-02 |
| NM_145825 | *Cetn4* | centrin 4 | 2.5E-01 | 3.6E-02 |
| NM_001110315 | *Kif1a* | kinesin family member 1A | 2.5E-01 | 2.5E-02 |
| NM_001029978 | *Tceal3* | transcription elongation factor A (SII)-like 3 | 2.5E-01 | 1.8E-02 |
| NM_011196 | *Ptger3* | prostaglandin E receptor 3 (subtype EP3) | 2.5E-01 | 2.5E-02 |
| NM_001177899 | *Skap1* | src family associated phosphoprotein 1 | 2.5E-01 | 4.3E-02 |
| NM_001004159 | *Clec4b2* | C-type lectin domain family 4, member b2 | 2.5E-01 | 1.1E-02 |
| NM_133203 | *Klra17* | killer cell lectin-like receptor, subfamily A, member 17 | 2.5E-01 | 2.6E-02 |
| NM_178907 | *Mapkapk3* | mitogen-activated protein kinase-activated protein kinase 3 | 2.5E-01 | 2.5E-02 |
| NM_001161798 | *Mthfr* | 5,10-methylenetetrahydrofolate reductase | 2.5E-01 | 3.3E-02 |
| NM_175752 | *Chn1* | chimerin 1 | 2.5E-01 | 4.9E-02 |
| NM_001159562 | *Il1rn* | interleukin 1 receptor antagonist | 2.5E-01 | 3.3E-02 |
| NM_001252484 | *Inca1* | inhibitor of CDK, cyclin A1 interacting protein 1 | 2.6E-01 | 3.7E-02 |
| NM_001081323 | *Mphosph9* | M-phase phosphoprotein 9 | 2.6E-01 | 4.9E-02 |
| NM_007976 | *F5* | coagulation factor V | 2.6E-01 | 1.1E-02 |
| NM_001007596 | *Rtn1* | reticulon 1 | 2.6E-01 | 3.5E-02 |
| NM_010071 | *Dok2* | docking protein 2 | 2.6E-01 | 2.6E-02 |
| NM_001177347 | *1700092M07Rik* | RIKEN cDNA 1700092M07 gene | 2.6E-01 | 3.8E-02 |
| NM_175860 | *Gimap1* | GTPase, IMAP family member 1 | 2.6E-01 | 4.7E-02 |
| NM_009678 | *Ap1m2* | adaptor protein complex AP-1, mu 2 subunit | 2.6E-01 | 3.3E-02 |
| NM_001253870 | *Tmem205* | transmembrane protein 205 | 2.6E-01 | 3.8E-02 |
| NM_177841 | *4932418E24Rik* | RIKEN cDNA 4932418E24 gene | 2.6E-01 | 3.3E-02 |
| NM_001164704 | *Renbp* | renin binding protein | 2.7E-01 | 3.3E-02 |
| NM_001109753 | *Sv2b* | synaptic vesicle glycoprotein 2 b | 2.8E-01 | 1.7E-02 |
| NM_174853 | *Disc1* | disrupted in schizophrenia 1 | 2.8E-01 | 4.4E-02 |
| NM_177346 | *Gpr149* | G protein-coupled receptor 149 | 2.8E-01 | 4.1E-02 |
| NM_001243067_dup1 | *Zfp433* | RIKEN cDNA 1700123A16 gene | 2.8E-01 | 3.8E-02 |
| NM_001286973 | *Dnajc19* | DnaJ (Hsp40) homolog, subfamily C, member 19 | 2.8E-01 | 3.3E-02 |
| NM_183147 | *Sprn* | shadow of prion protein | 2.8E-01 | 2.4E-02 |
| NM_001122595 | *A630033H20Rik* | RIKEN cDNA A630033H20 gene | 2.9E-01 | 3.3E-02 |
| NM_008213 | *Hand1* | heart and neural crest derivatives expressed transcript 1 | 2.9E-01 | 3.5E-02 |
| NR_040356 | *BB557941* | expressed sequence BB557941 | 2.9E-01 | 3.6E-02 |
| NM_019928 | *Klk4* | kallikrein related-peptidase 4 (prostase, enamel matrix, prostate) | 2.9E-01 | 2.8E-02 |
| NM_026739 | *9530077C05Rik* | RIKEN cDNA 9530077C05 gene | 2.9E-01 | 1.1E-02 |
| NM_008791 | *Pcp4* | Purkinje cell protein 4 | 2.9E-01 | 3.4E-02 |
| NM_001105196 | *Tfe3* | transcription factor E3 | 2.9E-01 | 3.8E-02 |
| NM_007710 | *Ckm* | creatine kinase, muscle | 2.9E-01 | 3.0E-02 |
| NM_001048054 | *Dusp16* | dual specificity phosphatase 16 | 3.0E-01 | 3.8E-02 |
| NR_045059 | *4930431P03Rik* | RIKEN cDNA 4930431P03 gene | 3.0E-01 | 4.3E-02 |
| NR_028572 | *Snora43* | - | 3.0E-01 | 3.6E-02 |
| NM_025355 | *Tceal6* | transcription elongation factor A (SII)-like 6 | 3.0E-01 | 4.2E-02 |
| NM_001243857 | *Fam124a* | family with sequence similarity 124, member A | 3.0E-01 | 4.3E-02 |
| NR_015589 | *9330158H04Rik* | RIKEN cDNA 9330158H04 gene | 3.0E-01 | 4.1E-02 |
| NM_007495 | *Astn1* | astrotactin 1 | 3.0E-01 | 3.3E-02 |
| NM_001256522 | *C330006A16Rik* | RIKEN cDNA C330006A16 gene | 3.0E-01 | 3.8E-02 |
| NM_001110497 | *Tmem87a* | transmembrane protein 87A | 3.0E-01 | 2.9E-02 |
| NM_001286607 | *Foxp2* | forkhead box P2 | 3.0E-01 | 3.3E-02 |
| NM_001159956 | *Pde1c* | phosphodiesterase 1C | 3.0E-01 | 4.0E-02 |
| NM_139138 | *Emr4* | EGF-like module containing, mucin-like,  hormone receptor-like sequence 4 | 3.0E-01 | 1.1E-02 |
| NM_001037759 | *Sgk3* | serum/glucocorticoid regulated kinase 3 | 3.0E-01 | 3.5E-02 |
| NM_008973 | *Ptn* | pleiotrophin | 3.0E-01 | 3.8E-02 |
| NR_040277 | *Plxna4os1* | plexin A4, opposite strand 1 | 3.1E-01 | 3.3E-02 |
| NM_199473 | *Col8a2* | collagen, type VIII, alpha 2 | 3.1E-01 | 3.5E-02 |
| NM_010160 | *Celf2* | CUGBP, Elav-like family member 2 | 3.1E-01 | 2.3E-02 |
| NM_001110160 | *Lrrc61* | leucine rich repeat containing 61 | 3.1E-01 | 3.8E-02 |
| NM_001252521 | *Picalm* | phosphatidylinositol binding clathrin assembly protein | 3.1E-01 | 4.7E-02 |
| NM_001252523 | *Picalm* | phosphatidylinositol binding clathrin assembly protein | 3.1E-01 | 4.0E-02 |
| NM_001012623_dup1 | *Rims1* | regulating synaptic membrane exocytosis 1 | 3.1E-01 | 2.6E-02 |
| NM_152923 | *Kcnq3* | potassium voltage-gated channel, subfamily Q, member 3 | 3.1E-01 | 3.8E-02 |
| NM_010927 | *Nos2* | nitric oxide synthase 2, inducible | 3.2E-01 | 1.2E-02 |
| NM_177759 | *Ccdc60* | coiled-coil domain containing 60 | 3.2E-01 | 4.3E-02 |
| NM_001146058 | *Acot7* | acyl-CoA thioesterase 7 | 3.2E-01 | 2.9E-02 |
| NM_001271772 | *Skil* | SKI-like | 3.2E-01 | 4.9E-02 |
| NM_009257 | *Serpinb5* | serine (or cysteine) peptidase inhibitor, clade B, member 5 | 3.2E-01 | 3.3E-02 |
| NM_173731 | *Hmgcll1* | 3-hydroxymethyl-3-methylglutaryl-Coenzyme A lyase-like 1 | 3.2E-01 | 4.1E-02 |
| NM_001285807 | *Dtna* | dystrobrevin alpha | 3.2E-01 | 2.2E-02 |
| NM_198297 | *Trat1* | T cell receptor associated transmembrane adaptor 1 | 3.3E-01 | 3.3E-02 |
| NM_001099299 | *Ajap1* | adherens junction associated protein 1 | 3.3E-01 | 4.1E-02 |
| NM_001286347 | *Eml1* | echinoderm microtubule associated protein like 1 | 3.3E-01 | 3.1E-02 |
| NM_019790 | *Tmeff2* | transmembrane protein with EGF-like and two follistatin-like domains 2 | 3.3E-01 | 4.8E-02 |
| NM_029004 | *Rasgef1c* | RasGEF domain family, member 1C | 3.3E-01 | 3.1E-02 |
| NR_037959 | *1600010M07Rik* | RIKEN cDNA 1600010M07 gene | 3.3E-01 | 1.1E-02 |
| NM_177713 | *Gapt* | Grb2-binding adaptor, transmembrane | 3.3E-01 | 1.9E-02 |
| NM_001081349 | *Slc43a1* | solute carrier family 43, member 1 | 3.3E-01 | 3.9E-02 |
| NM_001252655 | *Kcnab2* | potassium voltage-gated channel, shaker-related subfamily,  beta member 2 | 3.3E-01 | 4.4E-02 |
| NR_045331 | *Gm6307* | predicted gene 6307 | 3.3E-01 | 4.5E-02 |
| NR_040523 | *1700025N23Rik* | RIKEN cDNA 1700025N23 gene | 3.3E-01 | 4.9E-02 |
| NR_102723 | *Cd22* | CD22 antigen | 3.3E-01 | 3.1E-02 |
| NM_198864 | *Slitrk3* | SLIT and NTRK-like family, member 3 | 3.4E-01 | 3.8E-02 |
| NM_001163098 | *Tchh* | trichohyalin | 3.4E-01 | 4.7E-02 |
| NM_145741 | *Gdf10* | growth differentiation factor 10 | 3.4E-01 | 3.3E-02 |
| NM_001285530 | *Tle1* | transducin-like enhancer of split 1, homolog of Drosophila E(spl) | 3.4E-01 | 3.4E-02 |
| NM_153399 | *Syne1* | spectrin repeat containing, nuclear envelope 1 | 3.4E-01 | 2.6E-02 |
| NM_010004 | *Cyp2c40* | - | 3.4E-01 | 2.8E-02 |
| NM_010648 | *Klra3* | - | 3.5E-01 | 3.8E-02 |
| NM_001114179 | *Slc25a53* | solute carrier family 25, member 53 | 3.5E-01 | 5.0E-02 |
| NM_011800 | *Cdh20* | cadherin 20 | 3.5E-01 | 4.6E-02 |
| NM_183142 | *Alg11* | asparagine-linked glycosylation 11  (alpha-1,2-mannosyltransferase) | 3.5E-01 | 3.8E-02 |
| NM_013489 | *Cd84* | CD84 antigen | 3.5E-01 | 1.1E-02 |
| NM_001159648 | *Cntn1* | contactin 1 | 3.5E-01 | 3.3E-02 |
| NM_172994 | *Ppp2r2c* | protein phosphatase 2, regulatory subunit B, gamma | 3.5E-01 | 1.1E-02 |
| NM_001256520 | *Zfp672* | zinc finger protein 672 | 3.5E-01 | 4.9E-02 |
| NM_025506 | *Riiad1* | regulatory subunit of type II PKA R-subunit (RIIa) domain containing 1 | 3.5E-01 | 1.7E-02 |
| NM_001111331 | *Kcnip3* | Kv channel interacting protein 3, calsenilin | 3.5E-01 | 3.2E-02 |
| NM_177763 | *Lhfpl4* | lipoma HMGIC fusion partner-like protein 4 | 3.5E-01 | 1.7E-02 |
| NM_018797 | *Plxnc1* | plexin C1 | 3.5E-01 | 7.6E-03 |
| NM_013879 | *Cabp1* | calcium binding protein 1 | 3.6E-01 | 2.8E-02 |
| NM_001172205 | *Arid5a* | AT rich interactive domain 5A (MRF1-like) | 3.6E-01 | 1.1E-02 |
| NM_013462 | *Adrb3* | adrenergic receptor, beta 3 | 3.6E-01 | 3.1E-02 |
| NM_001160420 | *Optc* | opticin | 3.6E-01 | 4.8E-02 |
| NM_009711 | *Artn* | artemin | 3.6E-01 | 3.8E-02 |
| NM_001099644 | *Htr3a* | 5-hydroxytryptamine (serotonin) receptor 3A | 3.6E-01 | 2.8E-02 |
| NM_025655 | *Tmigd1* | transmembrane and immunoglobulin domain containing 1 | 3.6E-01 | 3.8E-02 |
| NM_008361 | *Il1b* | interleukin 1 beta | 3.6E-01 | 2.6E-02 |
| NR_040429 | *Gm15408* | - | 3.7E-01 | 4.8E-02 |
| NM_029425 | *4833424O15Rik* | RIKEN cDNA 4833424O15 gene | 3.7E-01 | 3.3E-02 |
| NM_007606 | *Car3* | carbonic anhydrase 3 | 3.7E-01 | 4.7E-02 |
| NM_172804 | *Syt16* | synaptotagmin XVI | 3.7E-01 | 4.5E-02 |
| NM_153098 | *Cd109* | CD109 antigen | 3.7E-01 | 1.1E-02 |
| NM_033268 | *Actn2* | actinin alpha 2 | 3.8E-01 | 3.0E-02 |
| NM_023893 | *Sapcd1* | suppressor APC domain containing 1 | 3.8E-01 | 4.8E-02 |
| NM_172460 | *Nphp3* | nephronophthisis 3 (adolescent) | 3.8E-01 | 1.8E-02 |
| NM_028116 | *Pygo1* | pygopus 1 | 3.8E-01 | 4.3E-02 |
| NM_175279 | *Rassf10* | Ras association (RalGDS/AF-6) domain family  (N-terminal) member 10 | 3.8E-01 | 3.3E-02 |
| NM_001205331 | *Map4* | microtubule-associated protein 4 | 3.8E-01 | 3.3E-02 |
| NM_007804 | *Cux2* | cut-like homeobox 2 | 3.8E-01 | 2.3E-02 |
| NM_016668 | *Bhmt* | betaine-homocysteine methyltransferase | 3.8E-01 | 4.9E-02 |
| NM_201639 | *Synm* | synemin, intermediate filament protein | 3.8E-01 | 3.3E-02 |
| NM_199021 | *Dpp10* | dipeptidylpeptidase 10 | 3.8E-01 | 4.5E-02 |
| NM_001039555 | *Cyp2c68* | cytochrome P450, family 2, subfamily c, polypeptide 68 | 3.8E-01 | 3.1E-02 |
| NM_001168572 | *Brsk1* | BR serine/threonine kinase 1 | 3.9E-01 | 3.3E-02 |
| NM_007581 | *Cacnb3* | calcium channel, voltage-dependent, beta 3 subunit | 3.9E-01 | 4.1E-02 |
| NM_010177 | *Fasl* | Fas ligand (TNF superfamily, member 6) | 3.9E-01 | 4.9E-02 |
| NM_016743 | *Nell2* | NEL-like 2 | 3.9E-01 | 3.5E-02 |
| NM_019992 | *Stap1* | signal transducing adaptor family member 1 | 3.9E-01 | 6.2E-03 |
| NM_207208 | *Clca6* | chloride channel calcium activated 6 | 3.9E-01 | 4.5E-02 |
| NM_013607 | *Myh11* | myosin, heavy polypeptide 11, smooth muscle | 3.9E-01 | 3.0E-02 |
| NM_177382 | *Cyp2r1* | cytochrome P450, family 2, subfamily r, polypeptide 1 | 3.9E-01 | 3.3E-02 |
| NM_008492 | *Ldhb* | lactate dehydrogenase B | 3.9E-01 | 4.0E-02 |
| NM_001014399 | *Abi3bp* | ABI gene family, member 3 (NESH) binding protein | 3.9E-01 | 2.6E-02 |
| NM_019942 | *06-Sep* | septin 6 | 3.9E-01 | 3.4E-02 |
| NR_028115 | *Lilra6* | leukocyte immunoglobulin-like receptor, subfamily A  (with TM domain), member 6 | 4.0E-01 | 1.7E-02 |
| NM_130866 | *Olfr78* | olfactory receptor 78 | 4.0E-01 | 3.1E-02 |
| NM_001038999 | *Atp8a1* | ATPase, aminophospholipid transporter (APLT), class I,  type 8A, member 1 | 4.0E-01 | 3.8E-02 |
| NM_173422 | *Colec10* | collectin sub-family member 10 | 4.0E-01 | 3.4E-02 |
| NM_028747 | *0610012H03Rik* | - | 4.0E-01 | 3.3E-02 |
| NM_001030291 | *Enpp7* | ectonucleotide pyrophosphatase/phosphodiesterase 7 | 4.0E-01 | 3.8E-02 |
| NM_020568 | *Plin4* | perilipin 4 | 4.0E-01 | 4.8E-02 |
| NM_001252505 | *St6gal1* | beta galactoside alpha 2,6 sialyltransferase 1 | 4.0E-01 | 4.5E-02 |
| NM_010214 | *Fhl4* | four and a half LIM domains 4 | 4.0E-01 | 4.2E-02 |
| NM_001201322 | *4930415O20Rik* | RIKEN cDNA 4930415O20 gene | 4.0E-01 | 4.1E-02 |
| NM_172271 | *Slc6a17* | solute carrier family 6 (neurotransmitter transporter), member 17 | 4.0E-01 | 3.3E-02 |
| NM_001163589 | *Prph* | peripherin | 4.1E-01 | 4.2E-02 |
| NM_027633 | *Fancd2os* | Fancd2 opposite strand | 4.1E-01 | 3.3E-02 |
| NM_009133 | *Stmn3* | stathmin-like 3 | 4.1E-01 | 3.5E-02 |
| NM_145978 | *Pdlim2* | PDZ and LIM domain 2 | 4.1E-01 | 3.8E-02 |
| NM_009937 | *Colq* | collagen-like tail subunit (single strand of homotrimer) of asymmetric acetylcholinesterase | 4.1E-01 | 3.8E-02 |
| NM_172951 | *Sntg2* | syntrophin, gamma 2 | 4.1E-01 | 3.2E-02 |
| NM_009922 | *Cnn1* | calponin 1 | 4.1E-01 | 3.3E-02 |
| NM_145634 | *Cd300lf* | CD300 antigen like family member F | 4.1E-01 | 4.5E-02 |
| NM_001081425 | *Rbm24* | RNA binding motif protein 24 | 4.1E-01 | 3.3E-02 |
| NM_009610 | *Actg2* | actin, gamma 2, smooth muscle, enteric | 4.1E-01 | 3.3E-02 |
| NM_010402 | *Hand2* | heart and neural crest derivatives expressed transcript 2 | 4.2E-01 | 3.1E-02 |
| NM_008685 | *Nfe2* | nuclear factor, erythroid derived 2 | 4.2E-01 | 4.8E-02 |
| NM_015811 | *Rgs1* | regulator of G-protein signaling 1 | 4.2E-01 | 2.5E-02 |
| NM_010654 | *Klrd1* | killer cell lectin-like receptor, subfamily D, member 1 | 4.2E-01 | 2.5E-02 |
| NM_053195 | *Slc24a3* | solute carrier family 24  (sodium/potassium/calcium exchanger), member 3 | 4.2E-01 | 3.1E-02 |
| NM_001004154 | *Rragb* | Ras-related GTP binding B | 4.2E-01 | 2.6E-02 |
| NM_001243761 | *Ciita* | class II transactivator | 4.2E-01 | 3.4E-02 |
| NM_001033489 | *Rnf207* | ring finger protein 207 | 4.2E-01 | 3.9E-02 |
| NM_001277876 | *Tpm2* | tropomyosin 2, beta | 4.2E-01 | 3.4E-02 |
| NM_170689 | *Ank3* | ankyrin 3, epithelial | 4.2E-01 | 4.8E-02 |
| NM_207583 | *Brinp2* | bone morphogenic protein/retinoic acid inducible neural-specific 2 | 4.2E-01 | 5.0E-02 |
| NM_001164111 | *Nfatc1* | nuclear factor of activated T cells, cytoplasmic,  calcineurin dependent 1 | 4.2E-01 | 2.8E-02 |
| NM_001159662 | *Ppp1r16b* | protein phosphatase 1, regulatory (inhibitor) subunit 16B | 4.2E-01 | 3.6E-02 |
| NM_009393 | *Tnnc1* | troponin C, cardiac/slow skeletal | 4.2E-01 | 4.2E-02 |
| NM_013781 | *Sh2d3c* | SH2 domain containing 3C | 4.3E-01 | 1.9E-02 |
| NM_024285 | *Bves* | blood vessel epicardial substance | 4.3E-01 | 3.3E-02 |
| NM_207663 | *Synm* | synemin, intermediate filament protein | 4.3E-01 | 4.7E-02 |
| NM_001034168 | *Ank2* | ankyrin 2, brain | 4.3E-01 | 4.1E-02 |
| NM_175201 | *Rnf38* | ring finger protein 38 | 4.3E-01 | 3.4E-02 |
| NM_173401 | *Fbxo44* | F-box protein 44 | 4.3E-01 | 4.5E-02 |
| NR_004447 | *Gm5523* | glyceraldehyde-3-phosphate dehydrogenase pseudogene | 4.3E-01 | 4.3E-02 |
| NM_001111058 | *Cd33* | CD33 antigen | 4.3E-01 | 4.1E-02 |
| NM_008773 | *P2ry2* | purinergic receptor P2Y, G-protein coupled 2 | 4.3E-01 | 1.1E-02 |
| NM_013868 | *Hspb7* | heat shock protein family, member 7 (cardiovascular) | 4.3E-01 | 4.5E-02 |
| NM_001081173 | *Lrch2* | leucine-rich repeats and calponin homology (CH) domain containing 2 | 4.3E-01 | 3.9E-02 |
| NM_138305 | *Adcy3* | adenylate cyclase 3 | 4.3E-01 | 1.1E-02 |
| NM_008712 | *Nos1* | nitric oxide synthase 1, neuronal | 4.3E-01 | 4.6E-02 |
| NM_001099297 | *Duox1* | dual oxidase 1 | 4.3E-01 | 3.3E-02 |
| NM_021566 | *Jph2* | junctophilin 2 | 4.3E-01 | 3.3E-02 |
| NM_008741 | *Nsg2* | neuron specific gene family member 2 | 4.3E-01 | 3.4E-02 |
| NM_178934 | *Slc2a12* | solute carrier family 2 (facilitated glucose transporter), member 12 | 4.3E-01 | 3.8E-02 |
| NM_007906 | *Eef1a2* | eukaryotic translation elongation factor 1 alpha 2 | 4.3E-01 | 4.7E-02 |
| NM_010876 | *Ncf1* | neutrophil cytosolic factor 1 | 4.3E-01 | 2.2E-02 |
| NR_040339 | *Gm19897* | - | 4.3E-01 | 4.0E-02 |
| NM_027120 | *Nmrk2* | nicotinamide riboside kinase 2 | 4.3E-01 | 2.6E-02 |
| NM_177740 | *Rgma* | repulsive guidance molecule family member A | 4.3E-01 | 3.1E-02 |
| NM_001004193 | *Rhox8* | reproductive homeobox 8 | 4.3E-01 | 3.8E-02 |
| NM_010043 | *Des* | desmin | 4.4E-01 | 3.3E-02 |
| NM_009548 | *Rnf112* | ring finger protein 112 | 4.4E-01 | 4.5E-02 |
| NM_023279 | *Tubb3* | tubulin, beta 3 class III | 4.4E-01 | 3.3E-02 |
| NM_138310 | *Apobr* | apolipoprotein B receptor | 4.4E-01 | 1.1E-02 |
| NM_026056 | *Cap2* | CAP, adenylate cyclase-associated protein, 2 (yeast) | 4.4E-01 | 3.3E-02 |
| NM_021316 | *Cend1* | cell cycle exit and neuronal differentiation 1 | 4.4E-01 | 2.5E-02 |
| NM_001038619 | *Dnm3* | dynamin 3 | 4.4E-01 | 3.0E-02 |
| NM_007403 | *Adam8* | a disintegrin and metallopeptidase domain 8 | 4.4E-01 | 1.1E-02 |
| NM_001038698 | *Elavl4* | ELAV (embryonic lethal, abnormal vision, Drosophila)-like 4  (Hu antigen D) | 4.4E-01 | 2.6E-02 |
| NM_021431 | *Nudt11* | nudix (nucleoside diphosphate linked moiety X)-type motif 11 | 4.4E-01 | 3.3E-02 |
| NR_045444 | *1700071M16Rik* | - | 4.4E-01 | 3.4E-02 |
| NM_148946 | *Slc8a2* | solute carrier family 8 (sodium/calcium exchanger), member 2 | 4.4E-01 | 3.1E-02 |
| NM_008083 | *Gap43* | growth associated protein 43 | 4.4E-01 | 2.6E-02 |
| NM_001282045 | *Schip1* | schwannomin interacting protein 1 | 4.4E-01 | 4.8E-02 |
| NM_001285490 | *Nap1l4* | nucleosome assembly protein 1-like 4 | 4.4E-01 | 4.9E-02 |
| NM_174998 | *Hpcal4* | hippocalcin-like 4 | 4.4E-01 | 4.3E-02 |
| NM_001076679 | *Gm9733* | predicted gene 9733 | 4.5E-01 | 4.8E-02 |
| NM_001081652 | *Nacad* | NAC alpha domain containing | 4.5E-01 | 4.9E-02 |
| NM_010867 | *Myom1* | myomesin 1 | 4.5E-01 | 3.8E-02 |
| NM_011087 | *Pira1* | - | 4.5E-01 | 1.9E-02 |
| NM_007765 | *Crmp1* | collapsin response mediator protein 1 | 4.5E-01 | 1.9E-02 |
| NM_001029878 | *Lonrf2* | LON peptidase N-terminal domain and ring finger 2 | 4.5E-01 | 4.3E-02 |
| NM_001159572 | *4632428N05Rik* | RIKEN cDNA 4632428N05 gene | 4.5E-01 | 5.0E-02 |
| NM_134438 | *Gpr37l1* | G protein-coupled receptor 37-like 1 | 4.5E-01 | 3.3E-02 |
| NM_010208 | *Fgr* | Gardner-Rasheed feline sarcoma viral (Fgr) oncogene homolog | 4.5E-01 | 1.8E-02 |
| NM_001286981 | *Mkks* | McKusick-Kaufman syndrome | 4.5E-01 | 2.5E-02 |
| NM_010554 | *Il1a* | interleukin 1 alpha | 4.5E-01 | 3.8E-02 |
| NR_040660 | *LOC100504703* | - | 4.5E-01 | 3.3E-02 |
| NM_001177647 | *Sirpa* | signal-regulatory protein alpha | 4.5E-01 | 2.6E-02 |
| NM_001081981 | *Nfix* | nuclear factor I/X | 4.5E-01 | 3.1E-02 |
| NM_203491 | *Chrm2* | cholinergic receptor, muscarinic 2, cardiac | 4.5E-01 | 4.8E-02 |
| NM_001113514 | *Itga9* | integrin alpha 9 | 4.5E-01 | 3.8E-02 |
| NM_007697 | *Chl1* | cell adhesion molecule with homology to L1CAM | 4.5E-01 | 3.1E-02 |
| NM_172875 | *Adc* | arginine decarboxylase | 4.6E-01 | 2.6E-02 |
| NM_008176 | *Cxcl1* | chemokine (C-X-C motif) ligand 1 | 4.6E-01 | 4.9E-02 |
| NM_001081211 | *Ptafr* | platelet-activating factor receptor | 4.6E-01 | 3.3E-02 |
| NM_001134802 | *Apol7e* | - | 4.6E-01 | 3.3E-02 |
| NM_001205011 | *Mptx2* | - | 4.6E-01 | 2.7E-02 |
| NM_198111 | *Akap6* | A kinase (PRKA) anchor protein 6 | 4.6E-01 | 3.4E-02 |
| NM_206535 | *Cd200r2* | Cd200 receptor 2 | 4.6E-01 | 2.6E-02 |
| NR_045275 | *8430429K09Rik* | RIKEN cDNA 8430429K09 gene | 4.6E-01 | 3.5E-02 |
| NM_015743 | *Nr4a3* | nuclear receptor subfamily 4, group A, member 3 | 4.6E-01 | 3.8E-02 |
| NM_001081025 | *Maats1* | MYCBP-associated, testis expressed 1 | 4.6E-01 | 4.8E-02 |
| NM_183171 | *Fez1* | fasciculation and elongation protein zeta 1 (zygin I) | 4.6E-01 | 4.3E-02 |
| NM_009308 | *Syt4* | synaptotagmin IV | 4.6E-01 | 4.9E-02 |
| NM_021361 | *Nova1* | neuro-oncological ventral antigen 1 | 4.6E-01 | 2.9E-02 |
| NM_011994 | *Abcd2* | ATP-binding cassette, sub-family D (ALD), member 2 | 4.6E-01 | 2.6E-02 |
| NM_025557 | *Pcp4l1* | Purkinje cell protein 4-like 1 | 4.6E-01 | 2.6E-02 |
| NM_010267 | *Gdap1* | ganglioside-induced differentiation-associated-protein 1 | 4.6E-01 | 3.3E-02 |
| NM_001172101 | *Rnh1* | ribonuclease/angiogenin inhibitor 1 | 4.7E-01 | 4.1E-02 |
| NM_198637 | *1700016K19Rik* | RIKEN cDNA 1700016K19 gene | 4.7E-01 | 4.8E-02 |
| NM_009662 | *Alox5* | arachidonate 5-lipoxygenase | 4.7E-01 | 2.6E-02 |
| NM_009204 | *Slc2a4* | solute carrier family 2 (facilitated glucose transporter), member 4 | 4.7E-01 | 4.8E-02 |
| NM_015822 | *Fbxl3* | F-box and leucine-rich repeat protein 3 | 4.7E-01 | 1.7E-02 |
| NM_145152 | *Lrrc3* | leucine rich repeat containing 3 | 4.7E-01 | 3.0E-02 |
| NR_026907 | *Wbscr25* | Williams Beuren syndrome chromosome region 25 (human) | 4.7E-01 | 3.5E-02 |
| NM_001113330 | *Crx* | cone-rod homeobox | 4.7E-01 | 4.9E-02 |
| NM_019925 | *Gpr132* | G protein-coupled receptor 132 | 4.7E-01 | 1.8E-02 |
| NM_008515 | *Lrrfip1* | leucine rich repeat (in FLII) interacting protein 1 | 4.7E-01 | 4.6E-02 |
| NR_033527 | *Ccl25* | chemokine (C-C motif) ligand 25 | 4.7E-01 | 3.1E-02 |
| NM_178675 | *Slc35f1* | solute carrier family 35, member F1 | 4.7E-01 | 2.5E-02 |
| NM_172815 | *Rspo2* | R-spondin 2 homolog (Xenopus laevis) | 4.7E-01 | 4.8E-02 |
| NM_001111021 | *Runx1* | runt related transcription factor 1 | 4.7E-01 | 4.9E-02 |
| NM_009140 | *Cxcl2* | chemokine (C-X-C motif) ligand 2 | 4.7E-01 | 5.0E-02 |
| NM_025285 | *Stmn2* | stathmin-like 2 | 4.7E-01 | 1.7E-02 |
| NM_172612 | *Rnd1* | Rho family GTPase 1 | 4.8E-01 | 4.9E-02 |
| NM_026769 | *Caly* | calcyon neuron-specific vesicular protein | 4.8E-01 | 4.9E-02 |
| NM_011616 | *Cd40lg* | CD40 ligand | 4.8E-01 | 4.3E-02 |
| NM_011350 | *Sema4f* | sema domain, immunoglobulin domain (Ig), TM domain, and short cytoplasmic domain | 4.8E-01 | 2.9E-02 |
| NM_080451 | *Synpo2* | synaptopodin 2 | 4.8E-01 | 2.9E-02 |
| NM_001164052 | *Pik3cd* | phosphatidylinositol 3-kinase catalytic delta polypeptide | 4.8E-01 | 1.8E-02 |
| NM_011401 | *Slc2a3* | solute carrier family 2 (facilitated glucose transporter), member 3 | 4.8E-01 | 1.1E-02 |
| NM_016719 | *Grb14* | growth factor receptor bound protein 14 | 4.8E-01 | 2.5E-02 |
| NM_001285804 | *Chchd7* | coiled-coil-helix-coiled-coil-helix domain containing 7 | 4.8E-01 | 4.5E-02 |
| NM_001199105 | *Trp53inp1* | transformation related protein 53 inducible nuclear protein 1 | 4.8E-01 | 4.8E-02 |
| NM_013800 | *Barx2* | BarH-like homeobox 2 | 4.8E-01 | 2.9E-02 |
| NM_011859 | *Osr1* | odd-skipped related 1 (Drosophila) | 4.8E-01 | 4.9E-02 |
| NM_028627 | *Psd* | pleckstrin and Sec7 domain containing | 4.8E-01 | 3.7E-02 |
| NM_001164249 | *Tpm1* | tropomyosin 1, alpha | 4.8E-01 | 3.3E-02 |
| NM_033587 | *Pcdhga4* | - | 4.8E-01 | 2.9E-02 |
| NM_001099328 | *Zfp831* | zinc finger protein 831 | 4.8E-01 | 3.3E-02 |
| NM_023879 | *Rpgrip1* | retinitis pigmentosa GTPase regulator interacting protein 1 | 4.9E-01 | 4.1E-02 |
| NM_031881 | *Nedd4l* | neural precursor cell expressed, developmentally down-regulated gene 4-like | 4.9E-01 | 4.2E-02 |
| NM_001134697 | *Ctxn3* | cortexin 3 | 4.9E-01 | 3.6E-02 |
| NM_001145824 | *Hipk3* | homeodomain interacting protein kinase 3 | 4.9E-01 | 3.5E-02 |
| NM_022030 | *Sv2a* | synaptic vesicle glycoprotein 2 a | 4.9E-01 | 3.3E-02 |
| NM_028325 | *Zcchc12* | zinc finger, CCHC domain containing 12 | 4.9E-01 | 4.2E-02 |
| NM_001268286 | *Ptprc* | protein tyrosine phosphatase, receptor type, C | 4.9E-01 | 3.9E-02 |
| NM_001284427 | *Smtn* | smoothelin | 4.9E-01 | 3.7E-02 |
| NM_133219 | *Gcnt2* | glucosaminyl (N-acetyl) transferase 2, I-branching enzyme | 4.9E-01 | 2.6E-02 |
| NM_021344 | *Tesc* | tescalcin | 4.9E-01 | 1.8E-02 |
| NM_175493 | *Gpr68* | G protein-coupled receptor 68 | 4.9E-01 | 3.7E-02 |
| NR_045428 | *2410021H03Rik* | - | 4.9E-01 | 4.7E-02 |
| NM_138312 | *Fam172a* | family with sequence similarity 172, member A | 4.9E-01 | 4.9E-02 |
| NM_001024719 | *Cyp2c67* | cytochrome P450, family 2, subfamily c, polypeptide 67 | 4.9E-01 | 4.9E-02 |
| NM_176913 | *Dpep2* | dipeptidase 2 | 4.9E-01 | 2.6E-02 |
| NM_001173459 | *LOC100038947* | - | 4.9E-01 | 3.9E-02 |
| NM_008711 | *Nog* | noggin | 4.9E-01 | 4.1E-02 |
| NM_007420 | *Adrb2* | adrenergic receptor, beta 2 | 4.9E-01 | 2.0E-02 |
| NM_175692 | *Snhg11* | small nucleolar RNA host gene 11 | 4.9E-01 | 3.7E-02 |
| NM_011110 | *Pla2g5* | phospholipase A2, group V | 5.0E-01 | 4.5E-02 |
| NM_172118 | *Myl9* | myosin, light polypeptide 9, regulatory | 5.0E-01 | 3.5E-02 |
| NM_173781 | *Rab6b* | RAB6B, member RAS oncogene family | 5.0E-01 | 3.1E-02 |
| NM_177794 | *Tmem26* | transmembrane protein 26 | 5.0E-01 | 3.4E-02 |

**Table S2. List of upregulated genes in the small intestine of ΔdblGATA mice as determined by RNA-sequencing.** FDR indicates the false discovery rate.

| **GenBank accession no.** | **Symbol** | **Description** | **Fold change** | **FDR corrected *p*-value** |
| --- | --- | --- | --- | --- |
| NM_001163360 | *Fignl1* | fidgetin-like 1 | 1.2E+02 | 3.8E-02 |
| NM_001172136 | *Exog* | endo/exonuclease (5'-3'), endonuclease G-like | 7.3E+01 | 2.6E-02 |
| NM_001177888 | *Cacna1g* | calcium channel, voltage-dependent, T type, alpha 1G subunit | 4.7E+01 | 3.1E-02 |
| NM_001168538 | *Cdadc1* | cytidine and dCMP deaminase domain containing 1 | 4.6E+01 | 3.3E-02 |
| NM_001286033 | *Stx2* | syntaxin 2 | 4.2E+01 | 4.1E-02 |
| NM_001159567 | *Meis2* | Meis homeobox 2 | 2.9E+01 | 3.6E-02 |
| NM_001200038 | *1700021K19Rik* | RIKEN cDNA 1700021K19 gene | 2.7E+01 | 3.0E-03 |
| NR_105027 | *1700124L16Rik* | - | 2.5E+01 | 4.9E-02 |
| NM_019789 | *Kcnip3* | Kv channel interacting protein 3, calsenilin | 2.2E+01 | 6.2E-03 |
| NM_028905 | *4932438H23Rik* | RIKEN cDNA 4932438H23 gene | 2.1E+01 | 2.9E-02 |
| NR_045412 | *C030018K13Rik* | - | 1.4E+01 | 4.3E-02 |
| NM_019910 | *Dcpp1* | demilune cell and parotid protein 1 | 1.3E+01 | 4.9E-02 |
| NR_037981 | *Gm15217* | predicted gene 15217 | 1.1E+01 | 3.1E-02 |
| NR_036654 | *Gm6525* | ribosomal protein L36a pseudogene | 9.4E+00 | 4.8E-02 |
| NM_001168645 | *Slc6a18* | solute carrier family 6 (neurotransmitter transporter), member 18 | 7.4E+00 | 4.5E-02 |
| NM_027152 | *Cd164l2* | CD164 sialomucin-like 2 | 6.5E+00 | 4.9E-02 |
| NM_027026 | *Lrrc46* | leucine rich repeat containing 46 | 5.9E+00 | 3.8E-02 |
| NM_001044705 | *Zscan21* | zinc finger and SCAN domain containing 21 | 5.8E+00 | 4.1E-02 |
| NM_001146198 | *Nkx2-1* | NK2 homeobox 1 | 5.6E+00 | 3.1E-02 |
| NM_001166537 | *Hmga1* | high mobility group AT-hook 1 | 5.6E+00 | 4.5E-02 |
| NM_001122959 | *Rad54l* | RAD54 like (S. cerevisiae) | 5.6E+00 | 3.5E-02 |
| NM_001277231 | *Crkl* | v-crk sarcoma virus CT10 oncogene homolog (avian)-like | 4.9E+00 | 5.0E-02 |
| NM_001252528 | *Pqbp1* | polyglutamine binding protein 1 | 4.8E+00 | 3.5E-02 |
| NM_008572 | *Mcpt8* | mast cell protease 8 | 4.3E+00 | 3.4E-02 |
| NR_028299 | *1700084E18Rik* | RIKEN cDNA 1700084E18 gene | 4.2E+00 | 2.9E-02 |
| NM_001159422 | *Ccdc58* | coiled-coil domain containing 58 | 4.1E+00 | 3.8E-02 |
| NM_001285835 | *Nox4* | NADPH oxidase 4 | 4.0E+00 | 3.9E-02 |
| NM_001159719 | *Sept2* | septin 2 | 3.9E+00 | 3.8E-02 |
| NM_001163290 | *Adck3* | aarF domain containing kinase 3 | 3.8E+00 | 2.6E-02 |
| NR_045363 | *1700024F13Rik* | - | 3.8E+00 | 4.9E-02 |
| NM_001271543 | *Cnot7* | CCR4-NOT transcription complex, subunit 7 | 3.8E+00 | 3.8E-02 |
| NR_045601 | *Alg9* | asparagine-linked glycosylation 9 (alpha 1,2 mannosyltransferase) | 3.7E+00 | 4.5E-02 |
| NM_001244200 | *Pax6* | paired box 6 | 3.5E+00 | 4.9E-02 |
| NM_130905 | *Cd209e* | CD209e antigen | 3.2E+00 | 4.9E-02 |
| NM_133836 | *Il15ra* | interleukin 15 receptor, alpha chain | 3.1E+00 | 3.5E-02 |
| NM_025752 | *4933411K16Rik* | RIKEN cDNA 4933411K16 gene | 3.0E+00 | 3.5E-02 |
| NM_175153 | *Vwa9* | von Willebrand factor A domain containing 9 | 2.9E+00 | 3.8E-02 |
| NM_013474 | *Apoa2* | apolipoprotein A-II | 2.8E+00 | 3.3E-02 |
| NM_001253700 | *Elp5* | elongator acetyltransferase complex subunit 5 | 2.6E+00 | 5.0E-02 |
| NM_001037848 | *Cnot2* | CCR4-NOT transcription complex, subunit 2 | 2.6E+00 | 3.3E-02 |
| NM_009653 | *Alas2* | aminolevulinic acid synthase 2, erythroid | 2.5E+00 | 3.3E-02 |
| NR_033993 | *Dnajc24* | DnaJ (Hsp40) homolog, subfamily C, member 24 | 2.5E+00 | 3.1E-02 |
| NM_001199154 | *Sncaip* | synuclein, alpha interacting protein (synphilin) | 2.5E+00 | 3.8E-02 |
| NM_010184 | *Fcer1a* | Fc receptor, IgE, high affinity I, alpha polypeptide | 2.4E+00 | 4.9E-02 |
| NM_001164569 | *Rffl* | ring finger and FYVE like domain containing protein | 2.4E+00 | 3.9E-02 |
| NM_008220 | *Hbb-bt* | hemoglobin, beta adult t chain | 2.4E+00 | 4.1E-02 |
| NR_027915 | *1700120K04Rik* | RIKEN cDNA 1700120K04 gene | 2.3E+00 | 4.2E-02 |
| NM_178926 | *Vmac* | vimentin-type intermediate filament associated coiled-coil protein | 2.2E+00 | 4.9E-02 |
| NM_001037800 | *Cd209b* | CD209b antigen | 2.1E+00 | 4.9E-02 |
| NM_001163531 | *Tmem175* | transmembrane protein 175 | 2.1E+00 | 4.5E-02 |
| NM_178055 | *Dnajb2* | DnaJ (Hsp40) homolog, subfamily B, member 2 | 2.1E+00 | 1.1E-02 |
| NM_001159942 | *Plekhg1* | pleckstrin homology domain containing, family G  (with RhoGef domain) member 1 | 2.0E+00 | 4.7E-02 |

**Table S3. Gene Ontology (GO) analysis of the 379 downregulated genes in the small intestine of ΔdblGATA mice.**

| **Enrichment score** | **GO** | **Term** | **P value** |
| --- | --- | --- | --- |
| 2.92 | GO:0006936 | muscle contraction | 2.3E−05 |
| 2.81 | GO:0016337 | cell-cell adhesion | 7.0E−03 |
| 2.16 | GO:0005149 | interleukin-1 receptor  binding | 2.0E−02 |
| 2.08 | GO:0006954 | inflammatory response | 5.0E−03 |
| 1.71 | GO:0043005 | neuron projection | 2.0E−03 |
|  | GO:0030424 | axon | 1.5E−02 |
| 1.63 | GO:0031649 | heat generation | 3.0E−06 |
|  | GO:0001659 | temperature  homeostasis | 9.0E−06 |
|  | GO:0001660 | fever | 2.0E−03 |
| 1.46 | GO:0045202 | synapse | 3.0E−04 |
|  | GO:0006836 | neurotransmitter transport | 4.6E−02 |
| 1.4 | GO:0010518 | positive regulation of phospholipase activity | 3.1E−02 |
|  | GO:0060193 | positive regulation of lipase activity | 3.6E−02 |
| 1.35 | GO:0048666 | neuron development | 2.6E−02 |
|  | GO:0030182 | neuron differentiation | 3.8E−02 |
| 1.32 | GO:0006954 | inflammatory response | 5.0E−03 |
|  | GO:0006955 | immune response | 4.6E−02 |

**Table S4. Primer sequences for real-time PCR.**

| **Target gene** | **Primer sequence** | |
| --- | --- | --- |
| *Lpl* | Forward: 5′-GGCAAGCAACACAACCAG-3′  Reverse: 5′-GTTCTCCGATGTCCACCTC-3′ | |
| *Slc2a4* | Forward: 5′-GACCAACTAAGGGCGGGAG-3′  Reverse: 5′-AGGGTGAGTGAGGCATTTTC-3′ | |
| *Adipoq* | Forward: 5′-GTTCCCAATGTACCCATTCGC-3′  Reverse: 5′-TGTTGCAGTAGAACTTGCCAG-3′ | |
| *Lep* | Forward: 5′-ATTTCACACACGCAGTCGG-3′  Reverse: 5′-CAGCACATTTTGGGAAGGCA-3′ | |
| *Pparg* | Forward: 5′-GGTGCTCCAGAAGATGAC-3′  Reverse: 5′-TTCCTGCTAATACAAGTCCTT-3′ | |
| *Cav1* | Forward: 5′-GTCTACGATCTTCGGCATCC-3′  Reverse: 5′-GCTCTTGATGCACGGTACAA-3′ | |
| *Cav2* | Forward: 5′-ACAGGATACCCGCAATGAAG-3′  Reverse: 5′-ATCTGCAGCCATGCTCTCTT-3′ | |
| *Cd36* | Forward: 5′-AGGTCTATCTACGCTGTGTTC-3′  Reverse: 5′-TGGTTGTCTGGATTCTGGAG-3′ | |
| *Cidec* | Forward: 5′-CCAGAAGCCAACTAAGAAGAT-3′  Reverse: 5′-AGGTCATAGGAAAGCGAGTAT-3′ | |
| *Ccr3* | Forward: 5′-CAGTGCTTTTGGGTGTTTGTC-3′  Reverse: 5′-GATTTCTAGGGTCTGTGTGCC-3′ | |
| *Ccl11* | Forward: 5′-AGCTAGTCGGGAGAGCCTAC-3′  Reverse: 5′-AAGGAAGTGACCGTGAGCAG-3′ | |
| *Il1b* | Forward: 5′-GCAACTGTTCCTGAACTCAACT-3′  Reverse: 5′-ATCTTTTGGGGTCCGTCAACT-3′ | |
| *Il6* | Forward: 5′-TAGTCCTTCCTACCCCAATTTCC-3′  Reverse: 5′-TTGGTCCTTAGCCACTCCTTC-3′ | |
| *Tnf* | Forward: 5′-CCTGTAGCCCACGTCGTAG-3′  Reverse: 5′-GGGAGTAGACAAGGTACAACCC-3′ | |
| *Ifng* | Forward: 5′-ATGAACGCTACACACTGCATC-3′  Reverse: 5′-CCATCCTTTTGCCAGTTCCTC-3′ | |
| *Il4* | Forward: 5′-CTGTAGGGCTTCCAAGGTGCTTCG-3′  Reverse: 5′-CCATTTGCATGATGCTCTTTAGGC-3′ | |
| *Il5* | Forward: 5′-ACAAGCAATGAGACGATGAG-3′  Reverse: 5′-CCACGGACAGTTTGATTCTT-3′ | |
| *Il13* | Forward: 5′-CCTGGCTCTTGCTTGCCTT-3′  Reverse: 5′-GGTCTTGTGTGATGTTGCTCA-3′ | |
| *Retnlg* | Forward: 5′-TGCCAATCGAGATGACTGTCC-3′  Reverse: 5′-CCACAAGCACAACCAGTGAC-3′ | |
| *Cebpe* | Forward: 5′-AGGGAAGAAAGGAAGAGGTA-3′  Reverse: 5′-CTTCTCCGTCACCAACTC-3′ | |
| *Alox15* | Forward: 5′-GGCTCCAACAACGAGGTCTAC-3′  Reverse: 5′-AGGTATTCTGACACATCCACCTT-3′ | |
| *Serpinb2* | Forward: 5′-GCCGCTCAGAAGATAACGAG-3′  Reverse: 5′-TGGCCAATGTTGATGAGATG-3′ |  |
| *Slco4c1* | Forward: 5′-GAGAACCCGGCTTTCGTCC-3′  Reverse: 5′-CGAACCCCCATTCTGATTCCT-3′ |  |
| *Nlrp12* | Forward: 5′-GGATGGCCTCTATCGACTGTC-3′  Reverse: 5′-CCTCTGCAATCCCCAGGAATAA-3′ | |
| *Faim* | Forward: 5′-GAGCAGACTGTCGCTAACCAT-3′  Reverse: 5′-CTACGTCCCAAACAGCTACGA-3′ |  |
| *Drd2* | Forward: 5′-GTGAACAGGCGGAGAATGGA-3′  Reverse: 5′-TGGGAGGGATGGGGCTATAC-3′ |  |
| *Thsd1* | Forward: 5′-GACCCGAGTCCTCGGAGAAG-3′  Reverse: 5′-GAAACCCACTGACACCGTTC-3′ |  |
| *Arg1* | Forward: 5′-GCAGAGGTCCAGAAGAAT-3′  Reverse: 5′-GAGTGTTGATGTCAGTGTG-3′ |  |
| *Nos2* | Forward: 5′-GTTCTCAGCCCAACAATACA-3′  Reverse: 5′-GTGGACGGGTCGATGTCAC-3′ |  |

| *Cebpa* | Forward: 5′-GTGACAATGACCGCCTGC-3′  Reverse: 5′-TTGACCAAGGAGCTCTCAGG-3′ |
| --- | --- |
| *Acaca* | Forward: 5′-TGATTCTCAGTTCGGGCACT-3′  Reverse: 5′-TCACCCCGAATAGACAGCTC-3′ |
| *Fasn* | Forward: 5′-GATGACACCAGCTTTGCCAA-3′  Reverse: 5′-CAGTGAGTTGAGGACCAGGT-3′ |
| *Scd* | Forward: 5′-GCGATACACTCTGGTGCTCA-3′  Reverse: 5′-TATTCTCCCGGGATTGAATG-3′ |
| *Ptrf* | Forward: 5′-GATAAAGAAACTGGAGGTCAA-3′  Reverse: 5′-ACTTCATCCTGGTAGATCAT-3′ |
| *Agpat2* | Forward: 5′-CGTTCGGTCCTTCAAGTA-3′  Reverse: 5′- GATTAGAGATGATGACACAGG-3′ |
| *Bscl2* | Forward: 5′-TCCTTCTACTACTCCTACATGCC-3′  Reverse: 5′-GGCGGTGGAGGAATCACAG-3′ |
| *Lmna* | Forward: 5′-GGATGCTGAGAACAGGCTACA-3′  Reverse: 5′-GCTTGGCGGAGTATGTCTTTT-3′ |
| *Zmpste24* | Forward: 5′-GCATCGGTGGACGCTATGT-3′  Reverse: 5′-TGTGCTAGGAAGGTCTCCCAA-3′ |
| *Akt2* | Forward: 5′-ACGTGGTGAATACATCAAGACC-3′  Reverse: 5′-GCTACAGAGAAATTGTTCAGGGG-3′ |
| *Psmb8* | Forward: 5′-GTGCAGGTTGTATTATCTTCGGA-3′  Reverse: 5′-CGAGTCCCATTGTCATCTACG-3′ |
| *Plin1* | Forward: 5′-CAAGCACCTCTGACAAGGTTC-3′  Reverse: 5′-GTTGGCGGCATATTCTGCTG-3′ |
| *CXCL10* | Forward: 5′-ATTCCTGCAAGCCAATTTTGTC-3′  Reverse: 5′-CATCTCTTCTCACCCTTCTTTTTCA-3′ |
| *IDO* | Forward: 5′-CGAGAAAGAGTTGAGAAGTT-3′  Reverse: 5′-TGGAGAGTTGGCAGTAAG-3′ |
| *CD23* | Forward: 5′-GCGTGGGACTCAGATCGTG-3′  Reverse: 5′-GCTGTTTTAGACTCTGTGTGGTG-3′ |
| *CCL17* | Forward: 5′-CGGACCCCAACAACAAGAGA-3′  Reverse: 5′-CTCCCTCACTGTGGCTCTTC-3′ |
| *Gapdh* | Forward: 5′-CTGGTATGACAATGA ATACG-3′  Reverse: 5′-GCAGCGAACTTTATTGATGG-3′ |

| *GADPH* | Forward: 5′-CTGGGCTACACTGAGCACC-3′  Reverse: 5′-AAGTGGTCGTTGAGGGCAATG-3′ |
| --- | --- |

**Table S5. Primer sequences for reverse-transcription PCR.**

| **Target Gene** | **Primer sequence** |
| --- | --- |
| *Ccl11* | Forward: 5′-CACGGTCACTTCCTTCACG-3′  Reverse: 5′-TGGGGATCTTCTTACTGGTA-3′ |
| *Ccl24* | Forward: 5′-TGTGACCATCCCCTCATCTGC-3′  Reverse: 5′-AAACCTCGGTGCTATTGCGCG-3′ |
| *Il3* | Forward: 5′-GGATACCCACCGTTTAACCAG-3′  Reverse: 5′-ACAGGTTTACTCTCCGAAAG-3′ |
| *Il5* | Forward: 5′-TGCAAGAGTTCCTTGGTGT-3′  Reverse: 5′-GCACAGTTTTGTGGGGTT-3′ |
| *Csf2* | Forward: 5′-CATGCCTGTCACGTTGAATG-3′  Reverse: 5′-TGAAATTGCCCCGTAGAC-3′ |
| *Hprt* | Forward: 5′-GTAATGATCAGTCAACGGGGGAC-3′  Reverse: 5′-CCAGCAAGCTTGCAACCTTAACCA-3′ |

**Supplementary figures**

**Figure S1**

**
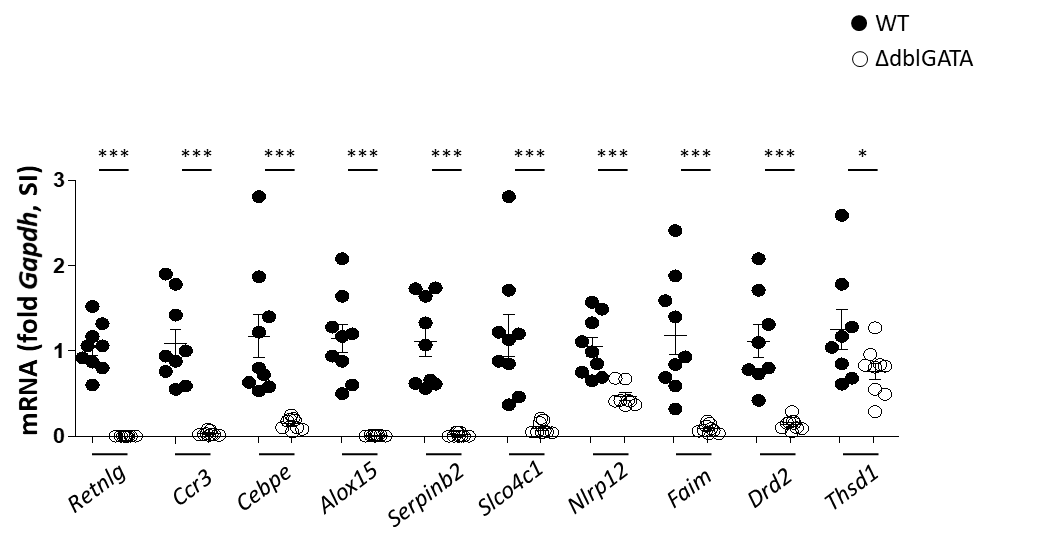
**

**Figure S2**

**
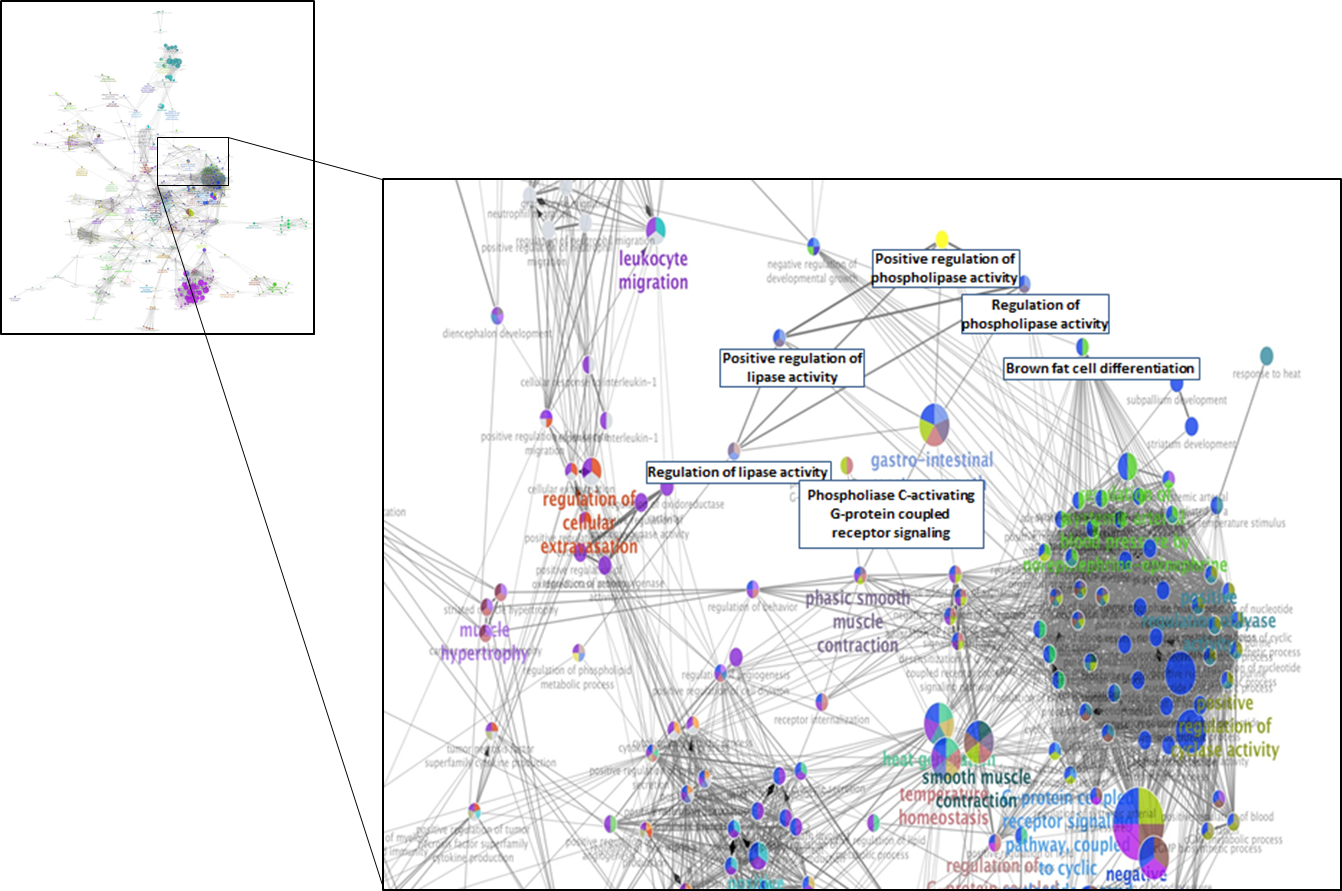
**

**Figure S3**

**
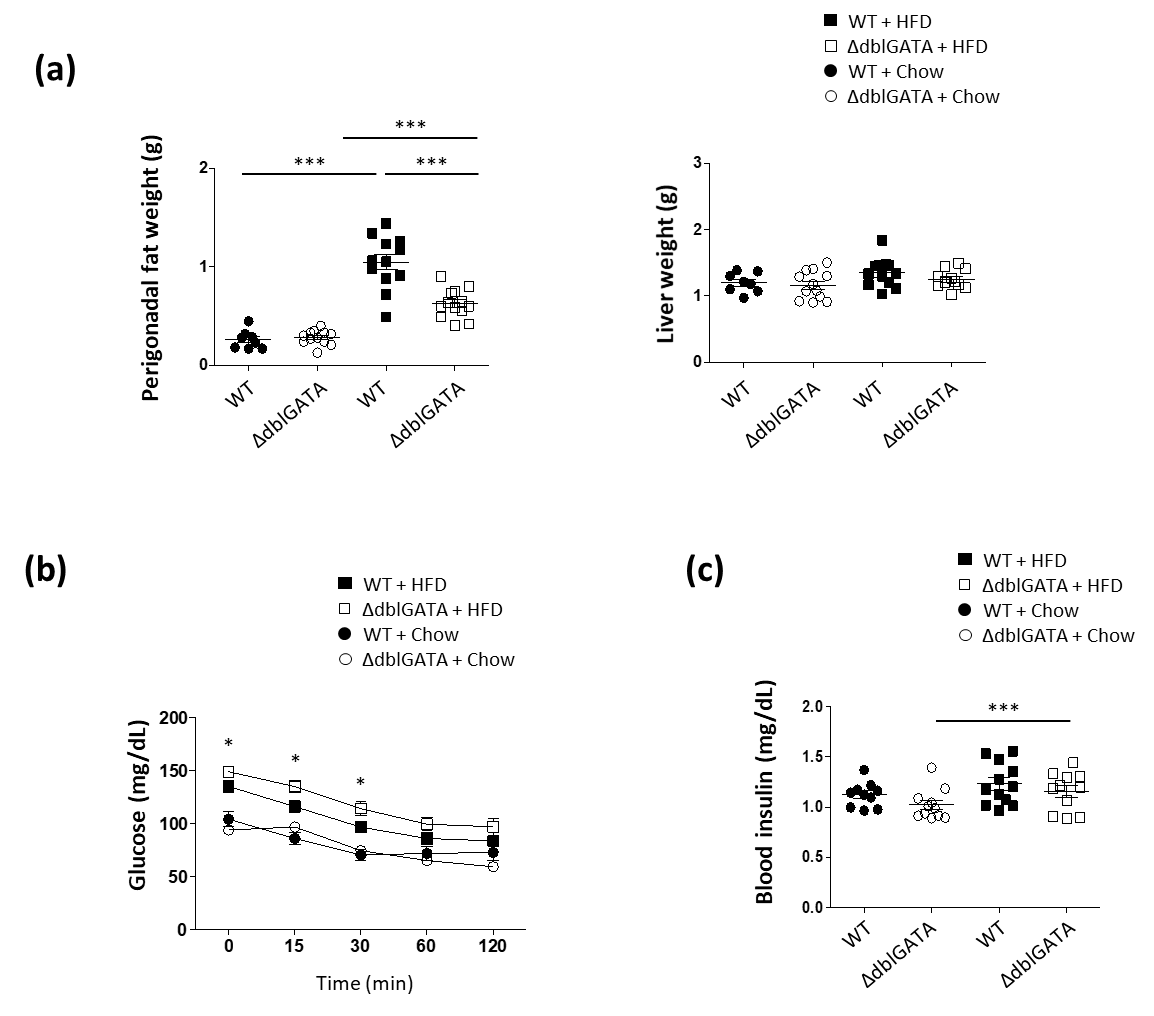
**

**Figure S4**

**
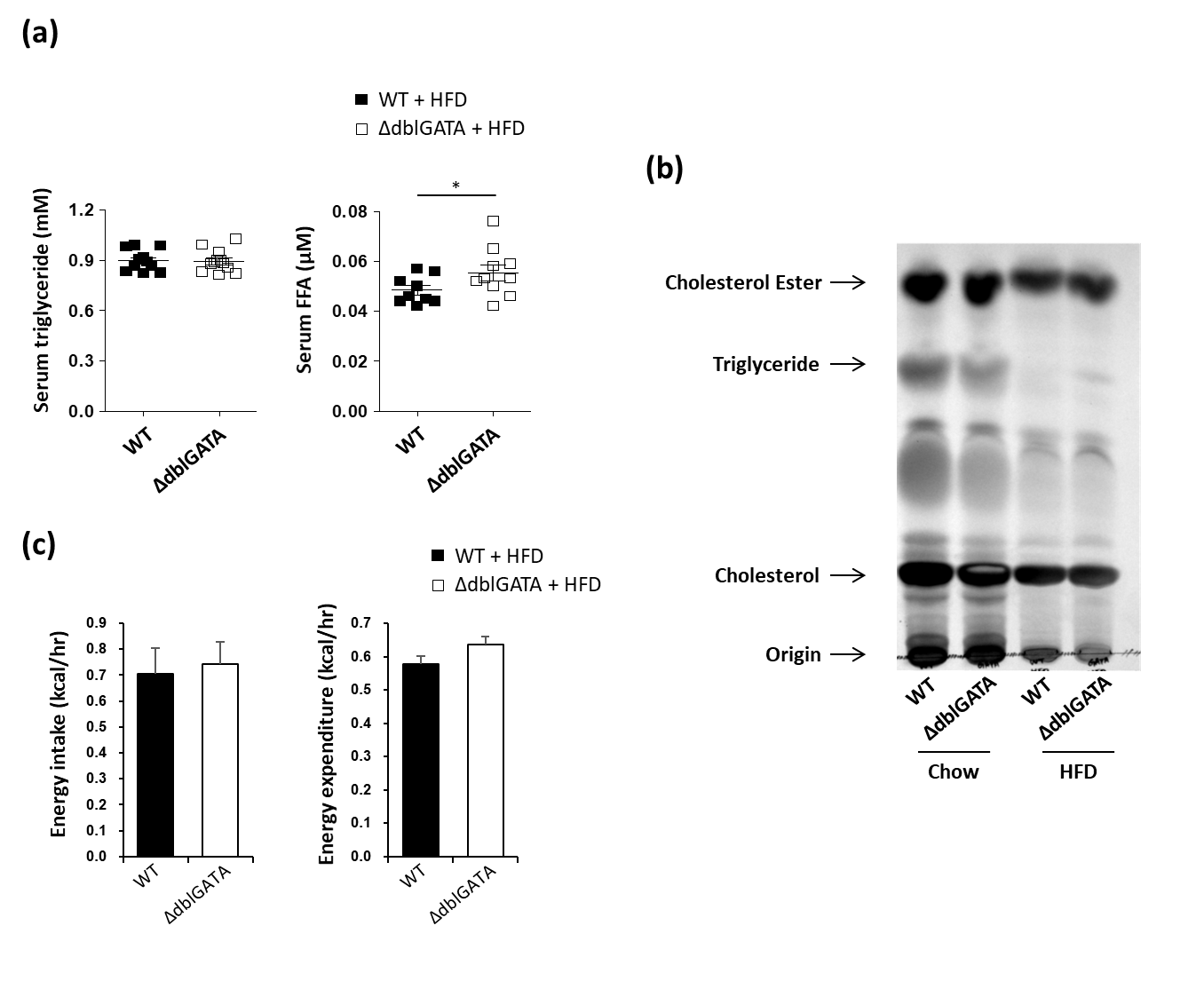
**

**Figure S5**

**
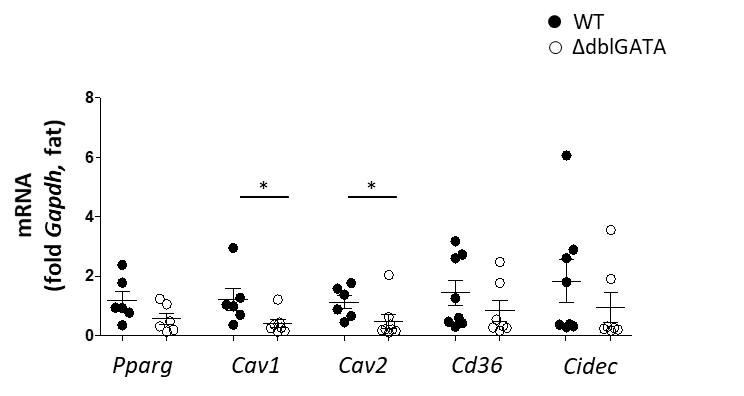
**

**Figure S6**

**
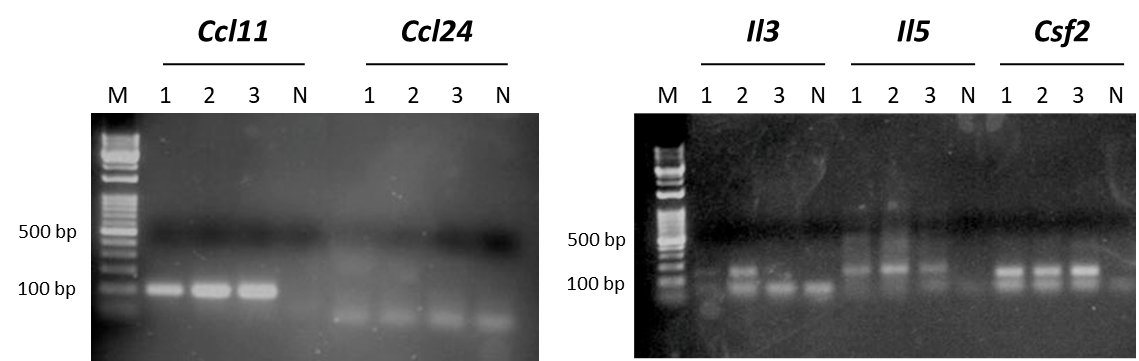
**

**Figure S7**

**
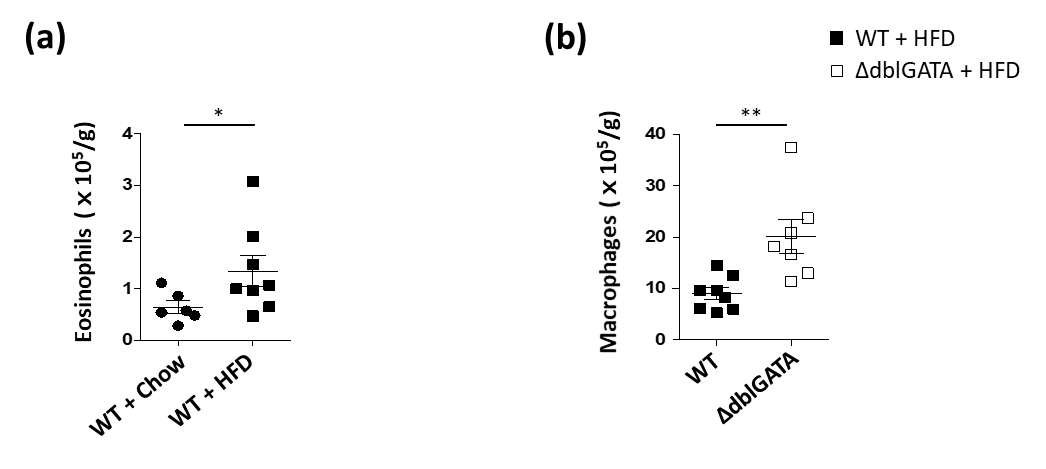
**

**Figure S8**

**
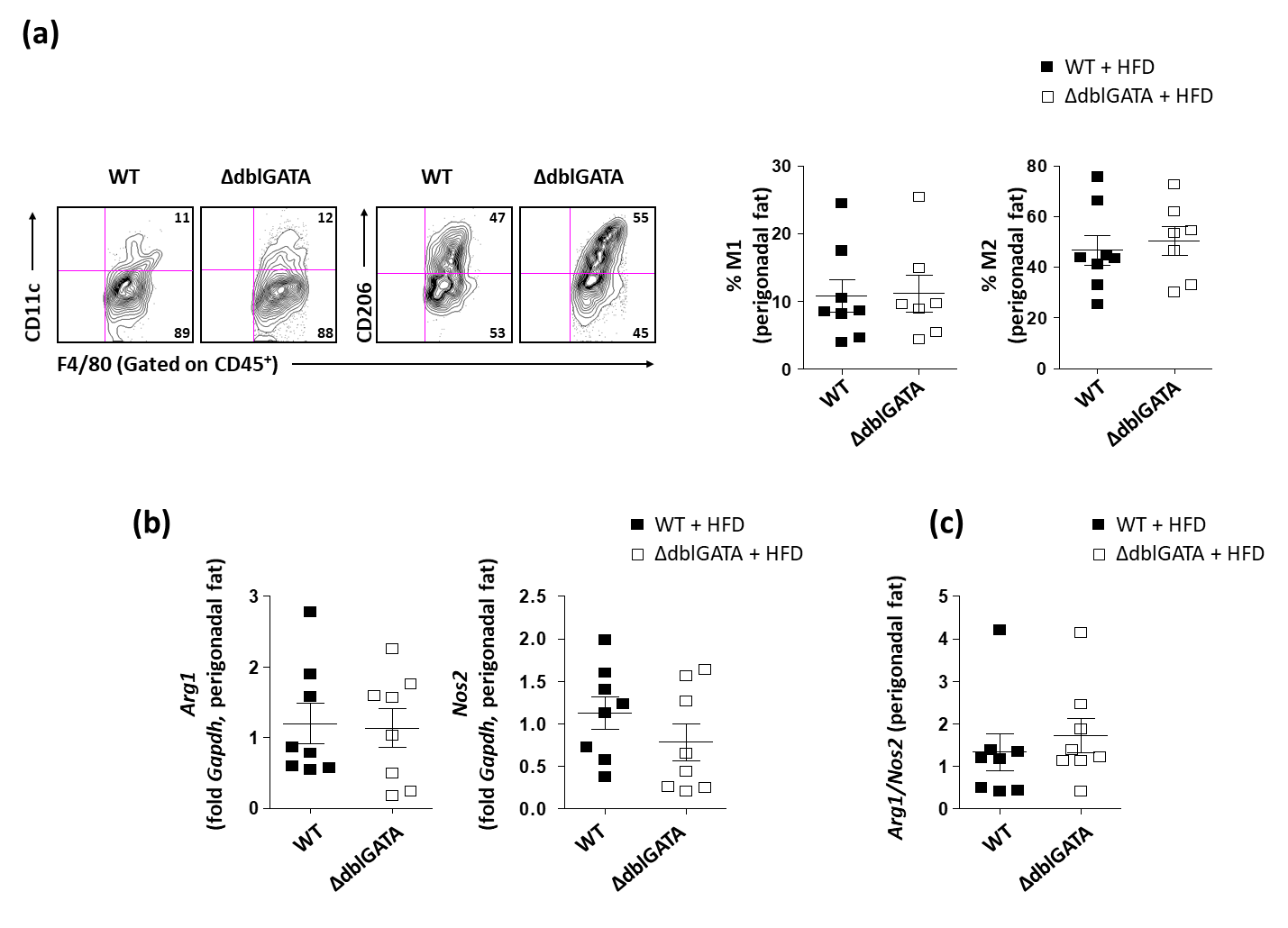
**

**Figure S9**

**
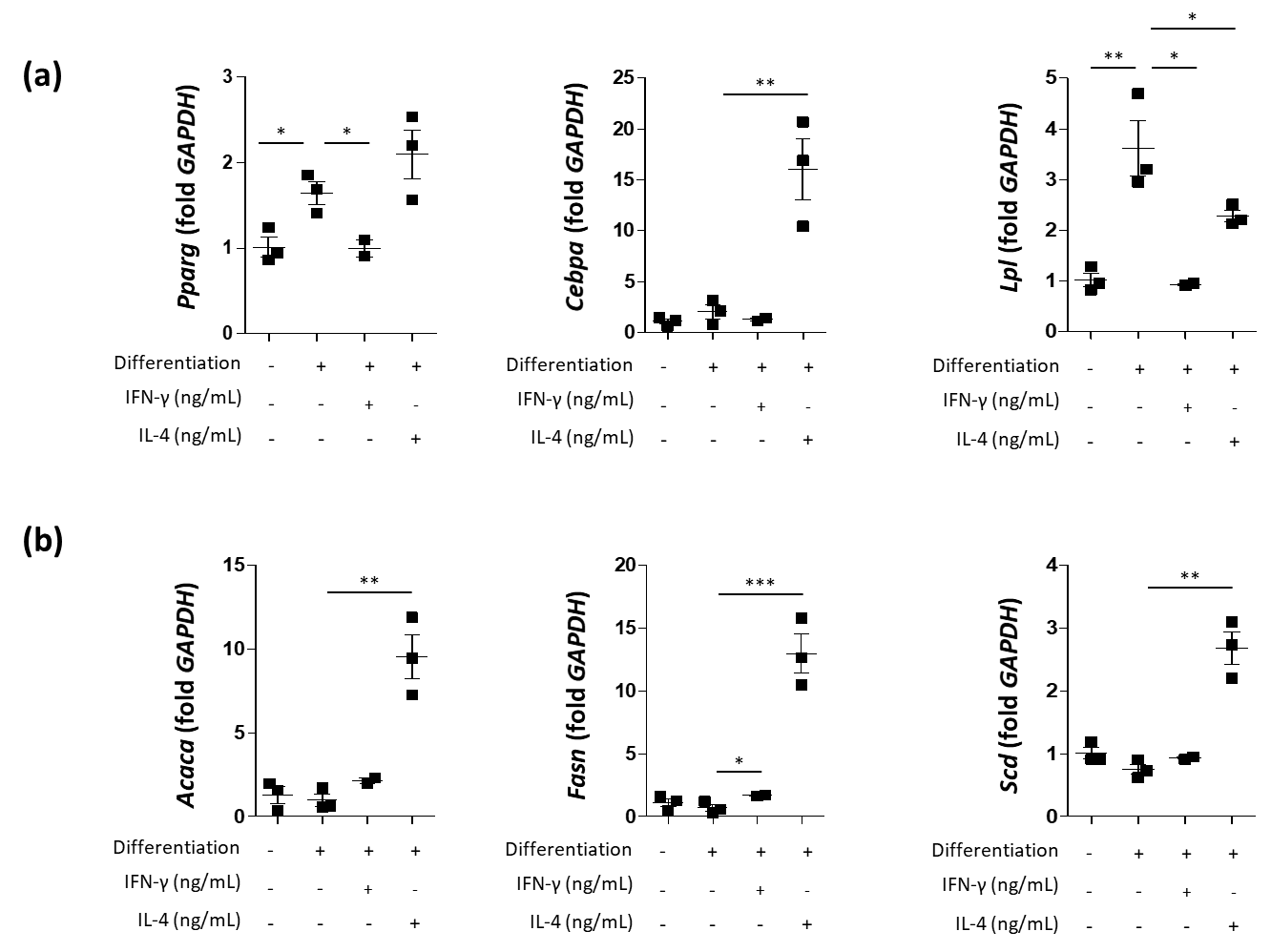
**

**Figure S10**

**
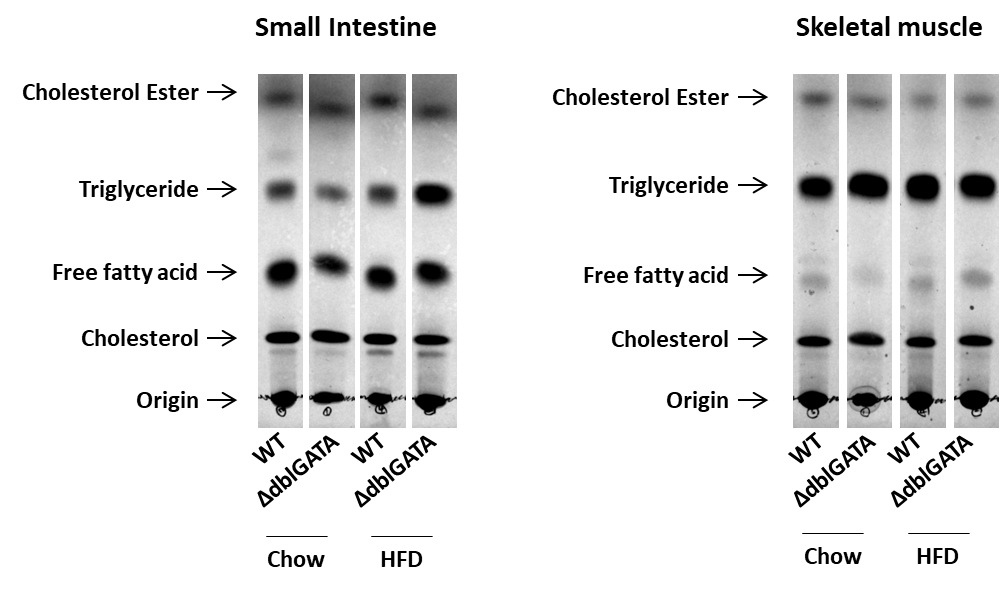
**

**Figure S11**

**
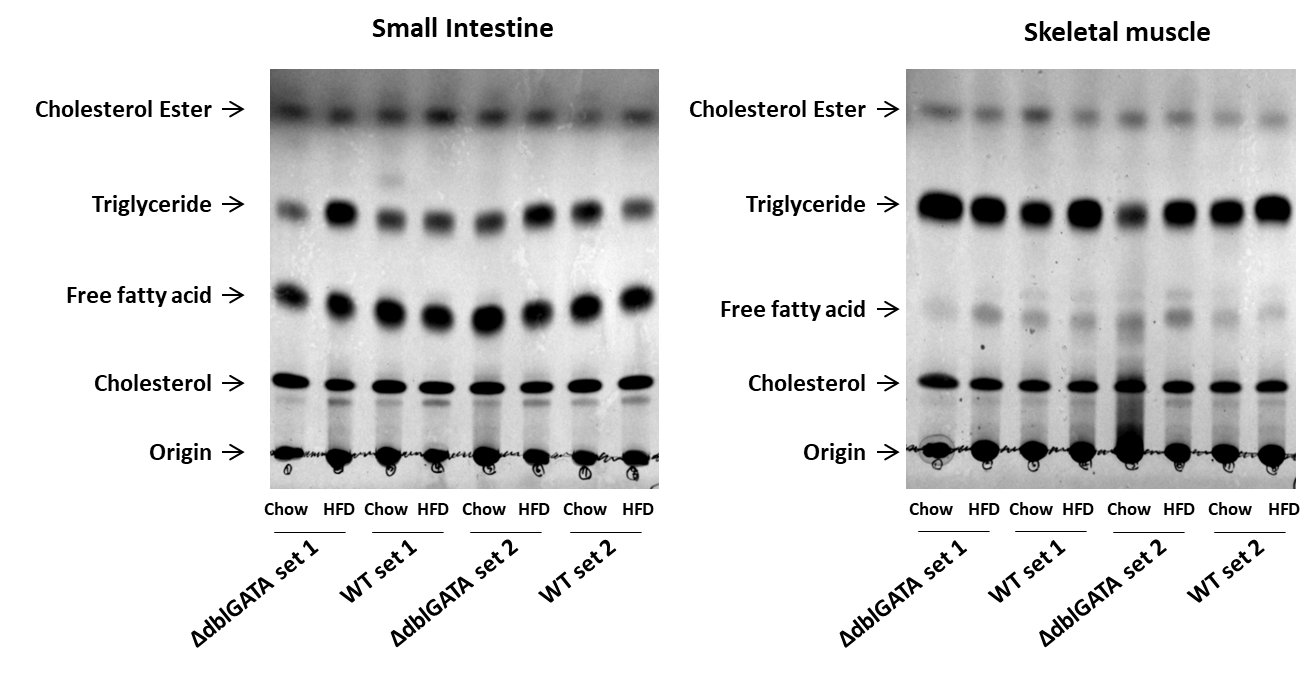
**

**Figure S12**

**
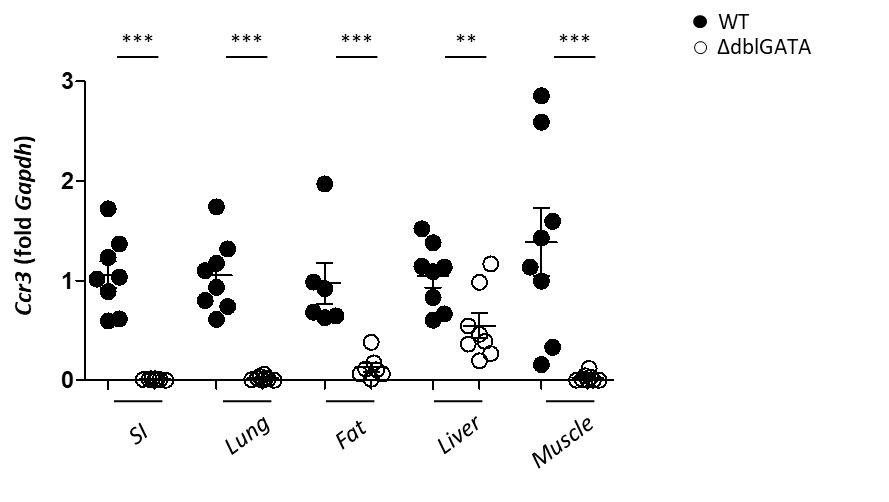
**

**Figure S13**

**
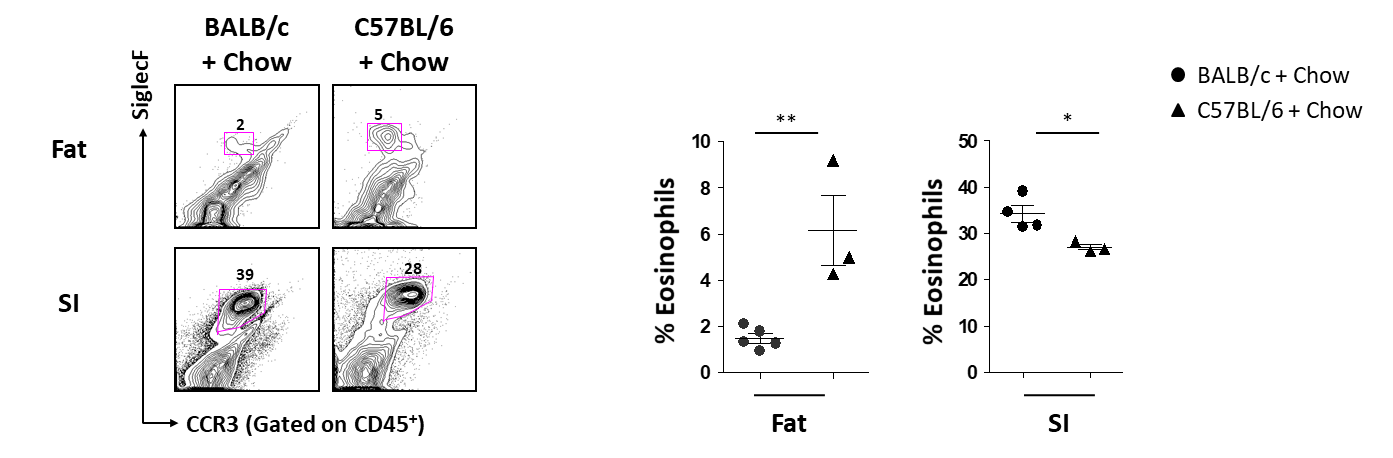
**

**Figure S14**

**
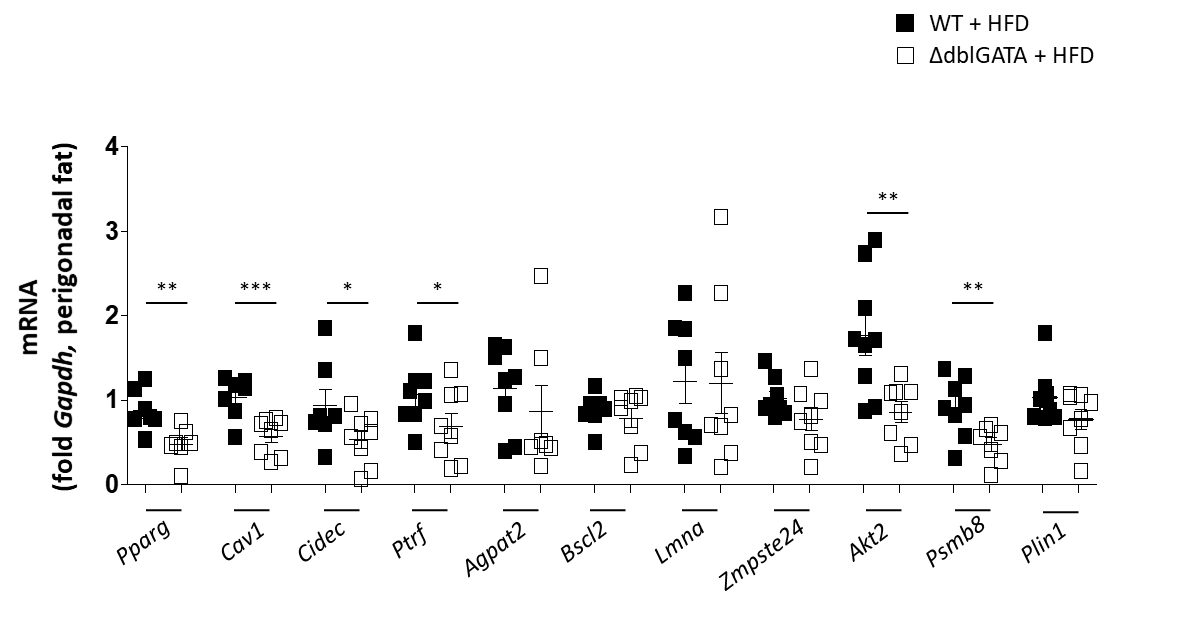
**

**Figure S15**

**
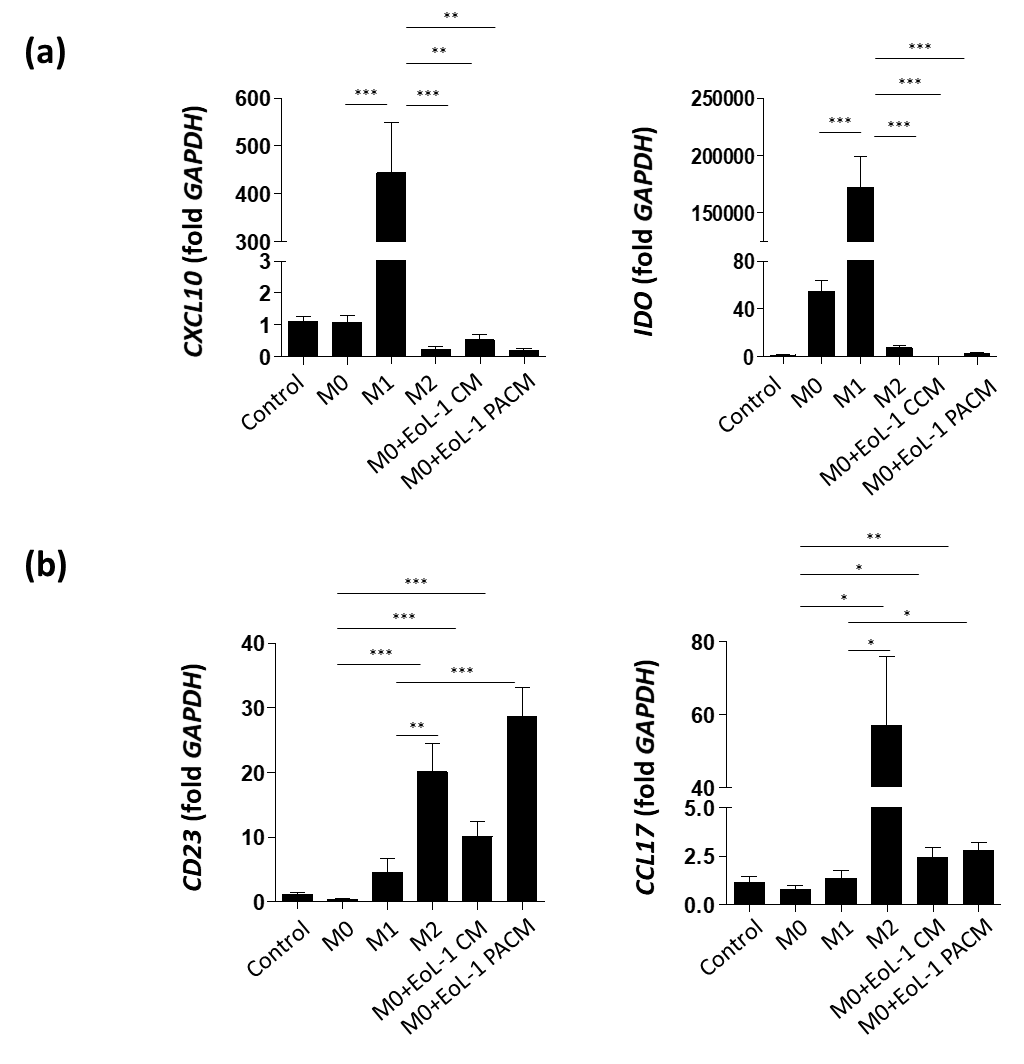
**

**Figure S16**

**
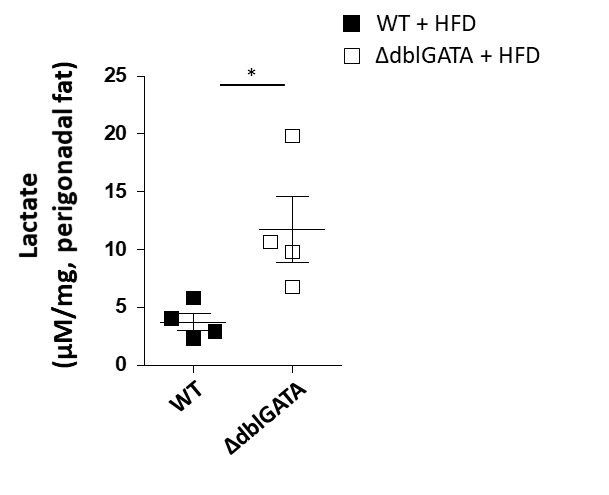
**
